# A human septin octamer complex sensitive to membrane curvature drives membrane deformation with a specific mesh-like organization

**DOI:** 10.1242/jcs.260813

**Published:** 2023-06-12

**Authors:** Koyomi Nakazawa, Gaurav Kumar, Brieuc Chauvin, Aurélie Di Cicco, Luca Pellegrino, Michael Trichet, Bassam Hajj, João Cabral, Anirban Sain, Stéphanie Mangenot, Aurélie Bertin

**Affiliations:** ^1^ Laboratoire Physico Chimie Curie, Institut Curie, PSL Research University, Sorbonne Université, CNRS UMR168, 75005, Paris, France; ^2^ Molecular Biophysics Unit, Indian Institute of Science Bangalore, Bangalore, Karnataka 560012, India; ^3^ Department of Chemical Engineering, Imperial College London, London SW7 2AZ, UK; ^4^ Sorbonne Université, CNRS, Institut de Biologie Paris-Seine (IBPS), Service de microscopie électronique (IBPS-SME), F-75005, Paris, France; ^5^ Department of Physics, Indian Institute of Technology Bombay, Powai, Mumbai 400076, India; ^6^ Laboratoire Matière et Systèmes Complexes (MSC), Université Paris Cité, CNRS UMR 7057, 75006 Paris, France

**Keywords:** Curvature, Cytoskeleton, Membrane, Septin

## Abstract

Septins are cytoskeletal proteins interacting with the inner plasma membrane and other cytoskeletal partners. Being key in membrane remodeling processes, they often localize at specific micrometric curvatures. To analyze the behavior of human septins at the membrane and decouple their role from other partners, we used a combination of bottom-up *in vitro* methods. We assayed their ultrastructural organization, their curvature sensitivity, as well as their role in membrane reshaping. On membranes, human septins organize into a two-layered mesh of orthogonal filaments, instead of generating parallel sheets of filaments observed for budding yeast septins. This peculiar mesh organization is sensitive to micrometric curvature and drives membrane reshaping as well. The observed membrane deformations together with the filamentous organization are recapitulated in a coarse-grained computed simulation to understand their mechanisms. Our results highlight the specific organization and behavior of animal septins at the membrane as opposed to those of fungal proteins.

## Teaser

**Highlighted Article:** The human octameric septin complex forms a two-layered mesh of orthogonal filaments on the membrane, which is sensitive to micrometric membrane curvature and drives membrane reshaping.

## INTRODUCTION

Septins are ubiquitous cytoskeletal protein complexes (Mostowy and Cossart, 2012) that interact directly with the inner plasma membranes (Bertin et al., 2010; Szuba et al., 2021; Zhang et al., 1999) as well as with actin and microtubules (Spiliotis, 2018) in eukaryotes. Septins are involved in essential cellular functions such as cell division (Cao et al., 2009; Surka et al., 2002; Tamborrini and Piatti, 2019), cell motility (Gilden et al., 2012) and cell compartmentalization (Caudron and Barral, 2009; Ewers et al., 2014; Hu et al., 2010). As septins have multiple functions, septin malfunction is the cause of several diseases (Camarda et al., 2017). Septin misregulation is thereby associated with the emergence of cancers and neurodegenerative disorders among other major illnesses (Hall and Russell, 2004). So far, the molecular organization and resulting mechanisms associated with the multiple observed functions of septins remain unknown.

In humans, 13 septin subunits are expressed in a tissue-dependent manner (Cao et al., 2007). They can assemble into linear palindromic complexes that are either hexameric or octameric (Martins et al., 2023). Hence, multiple septin subunit combinations can be observed. Human septins play many important roles in cells, particularly when associated to membranes. Indeed, human septin complexes often interact with membranes displaying micrometer-scale membrane curvatures (Cannon et al., 2017). They are found, assembling into ring-like structures, at the intercellular bridge connecting two dividing cells (Addi et al., 2018; Karasmanis et al., 2019), at the base of cilia (Hu et al., 2010), at the annulus of spermatozoa (Ihara et al., 2005) or at the base of dendrites (Tada et al., 2007). When bound to membranes, mammalian septins appear to control both the integrity and mechanical stability of membranes (Gilden et al., 2012; Patzig et al., 2016). Septins are thus likely involved in membrane remodeling.

Indeed, from our previous investigations focused on budding yeast septins (Beber et al., 2019b), we have shown that septins can deform membranes in a curvature-sensitive manner by directly interacting with membranes. In budding yeast, septins were shown to be essential for cell division and localized at the constriction sites between the mother cell and the bud (Tamborrini and Piatti, 2019). Investigations from our laboratory (Beber et al., 2019b) and from the Gladfelter laboratory (Cannon et al., 2017, 2019) have shown that septins are sensitive to micrometric curvatures both *in situ* and in cell-free assays. They thus differ from most curvature-sensing proteins, like bar proteins, which sense much sharper curvatures in nanometric ranges.

As opposed to yeast septins, which have been already thoroughly studied using bottom-up *in vitro* assays, the behavior of human septins at the membrane has not been examined in controlled environments *in vitro*, and barely been examined in cellular contexts (Martins et al., 2023). Indeed, until very recently, human septin octamers including the essential septin SEPT9 were not available for *in vitro* studies. A single study from 2009 (Tanaka-Takiguchi et al., 2009) using hexameric septins (deprived of SEPT9) showed that septin complexes could produce tubulation at the surface of giant unilamellar vesicles (GUVs), suggesting that human septins could remodel membranes. Structures from individual human septin subunits have also been generated (Castro et al., 2020). Recently, we expressed and purified human SEPT2 (SEPTIN2), SEPT6 (SEPTIN6), SEPT7 (SEPTIN7) and SEPT9_i1 (SEPTIN9 isoform 1) complexes recombinantly from *Escherichia coli* (Iv et al., 2021).

In the present work, we studied how human septin complexes can possibly remodel membranes. We used a combination of bottom-up *in vitro* assays at different scales using fluorescence and electron microscopy. Hence, the structural organization of septin filaments coupled with membrane remodeling could be probed.

We found that, similarly to budding yeast septins, human octameric septin complexes are curvature sensitive and remodel membranes. However, the nature of the observed deformations as well as the human septin organization contrast with the observations gathered using budding yeast septins. We show that human septin filaments indeed assemble into a network-like array interconnecting two layers of septin filaments, and the observed deformations display a curvature opposite to that induced by yeast septins. This distinct behavior reflects a peculiar and unique behavior of human septin complexes when bound to membranes. The observed membrane remodeling and filamentous organization was modeled by a coarse-grained simulation based on a nematic liquid crystalline 2D ordering of the septin filaments. The model confirmed that this specific behavior results from the mesh-size organization of human septins. Our observations thus suggest that the function of metazoan septins has significantly diverged from the role of fungal septins at the membrane.

## RESULTS

### Human septin filaments self-assemble on model lipid membranes

To characterize and visualize the polymerization and self-assembly of septins interacting with model lipid membranes, human septin octamers (SEPT2–GFP-SEPT6-SEPT7-SEPT9_i1-SEPT9_i1-SEPT7-SEPT6-SEPT2–GFP) were incubated with model lipid membranes. We first investigated whether negatively charged dioleoylphosphatidylserine (DOPS) and phosphatidylinositol 4,5-bisphosphate [PI(4,5)P_2_] could play a crucial role in the recruitment of septins to membranes using fluorescence microscopy. GUVs of different lipid compositions were generated and incubated with septins (see Materials and Methods). Septins exclusively interacted with membranes containing negatively charged lipids [DOPS (Fig. S1B) and/or PI(4,5)P_2_ (Fig. S1)]. Besides, the interaction was enhanced in the presence of both DOPS and PI(4,5)P_2_ (Fig. S1C), which resulted in enhanced recruitment of septins at the surface of GUVs. Interestingly, to detect any interaction between membranes and budding yeast septins, PI(4,5)P_2_ was required and DOPS was not sufficient (Beber et al., 2019a). In the following assays, artificial lipidic systems [small unilamellar vesicles (SUVs), large unilamellar vesicles (LUVs) or GUVs] thus contained both DOPS and PI(4,5)P_2_. The ionic strength was adjusted to induce septin polymerization, with 75 mM NaCl. Polymerization and further assembly of higher-order filaments occurred within minutes at room temperature.

**Fig. 1.**
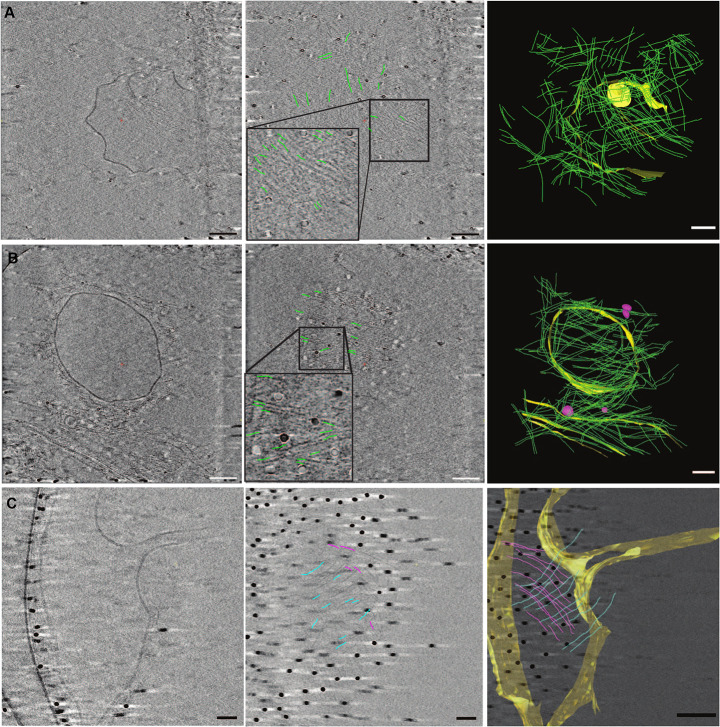
**Cryo-electron tomography of septins bound to liposomes.** (A–C) Slices of cryo-electron tomography (left and middle) and models (right) built by segmentation of tomograms. The left column displays slices in which the membrane is visible. The middle column displays slices in which one layer of the mesh is visualized. Septins bound to a deformed LUV (A) and a round LUV (B) are organized as mesh structures. The mesh consists of two layers of septins (C); the first (bound to membrane) and the second (bound to the first layer of septin filaments) layers of septin filaments are segmented in blue and pink, respectively, in the right image. In A,B, parts of some filaments are indicated using short green lines. Yellow and pink densities in the right columns represent membranes and septin filaments, respectively. Scale bars: 100 nm (left, middle); 200 nm (right).

To visualize both vesicles and septin filaments in three dimensions with nanometer resolution, we performed cryo-electron tomography. We observed the organization of septins bound to LUVs, which are small enough to be observed by transmission cryo-electron microscopy (cryo-EM). Indeed, GUVs, with a typical size in the micrometer range, are too thick to be suitable for observation by transmission cryo-EM. Slices of cryo-electron tomograms of representative LUVs decorated with septin filaments are shown in Fig. 1. On vesicles deformed by septins (about 50% of the liposomes), orthogonal mesh-like arrangements of septin filaments were observed, bound to membranes as highlighted by the segmentations (Fig. 1A–C, right panels). Interestingly, from the tomograms shown in Fig. 1 and Movie 1, those networks combined two layers of septins orthogonal to one another (the first and second layers are shown in blue and pink, respectively, in Fig. 1C, right). Movie 1 shows the consecutive *z*-slices from a 3D reconstruction going downwards in the *z*-axis and then upwards. The first filament layer (closest to the membrane) appears at t=2 s and t=31 s, within the sequence of images displayed in the movie, whereas the second layer appears at t=3 and 29 s, suggesting that the two filamentous layers are at distinct distances from the membrane. Comparing the distances at which the corresponding *z*-slices appear, the two sets of filaments are about 12 nm apart, taking into account the distance between the two *z*-slices. They are thereby close enough to interact through the flexible coiled coil domains of septins. The first layer of septin filaments appears to interact directly with the membrane, whereas the second layer appears to interact with the first layer of septins. Within the mesh, the distance between filaments was measured to be 18±15 nm (mean±s.d., *n*=157, seven cryo-tomograms; Fig. S2). Occasionally, membrane deformations were observed at nanometer scales (Fig. 1A). It is, however, not clear whether these nanometric deformations are caused by septin–membrane interactions. Indeed, perfectly spherical vesicles coated with septin meshes (Fig. 1B) were also observed.

### Septin filament networks on solid surfaces supported micrometric undulated membranes

**Fig. 2.**
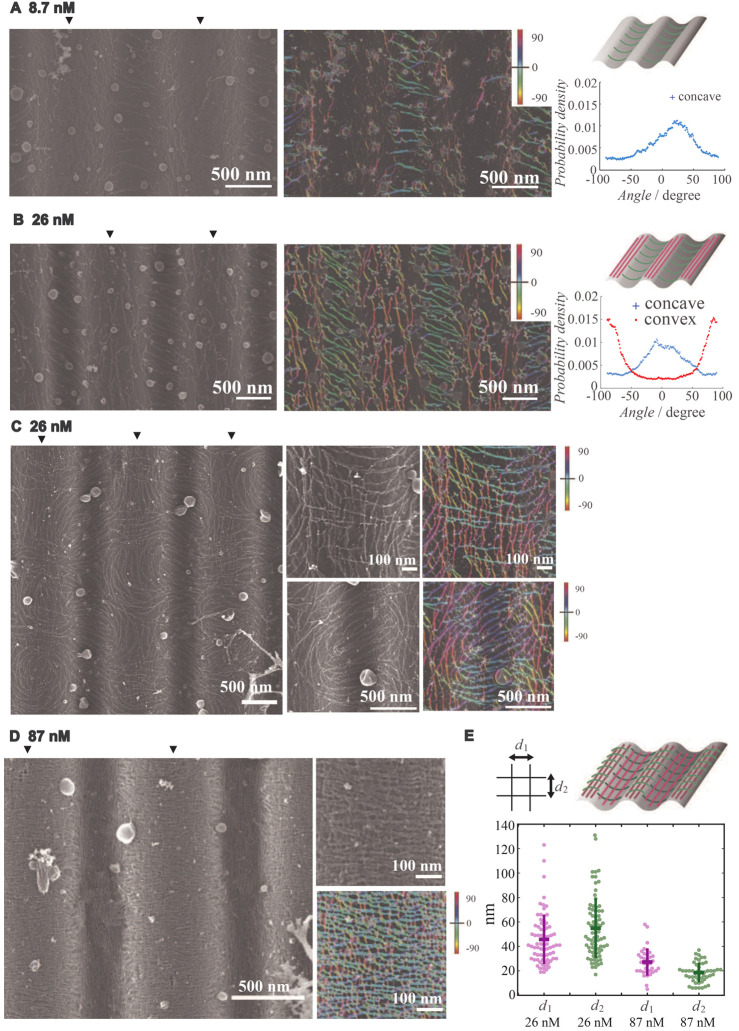
**SEM of septin filaments on lipid bilayers supported by undulated solid substrates.** Samples were prepared with different concentrations of septins dispersed in 10 mM Tris buffer containing 75 mM NaCl on undulated solid substrates with 1.6 µm periodicity and 0.20 µm amplitude. (A,B) SEM image of septin filaments prepared at (A) 8.7 nM and (B) 26 nM concentrations in solution. Raw SEM images are shown on the left. Black arrowheads show the top of convex hill of the substrate. Segmented filaments colored according to their orientations were overlaid on the raw SEM images and are shown in the middle. Distributions of orientation at the concave and convex parts of the substrates are plotted in the graphs on the right. Representations of the obtained results are shown in the schematics above the graphs. (C) SEM images of septin filaments at 26 nM concentration in solution. An image covering a wide range is shown on the left, and images focused on mesh structures that appeared on convex regions and the circular pattern of septin filaments on concave regions are shown in the top middle and bottom middle, respectively. The corresponding segmentation results of filaments colored according to their orientations are shown on the right. (D) SEM image of septin filaments at 87 nM concentration in solution. An image covering a wide range is shown on the left, and an image focused on mesh stru­ctures that appeared on the convex part of substrates is shown on the top right. Segmentation results of filaments colored according to their orientations are shown on the bottom right. (E) Measured distances between two adjacent septin filaments at the center of convex regions. Distances *d*_1_ and *d*_2_ correspond to the distances between two adjacent filaments in the direction parallel to the 1D wavy line of the substrate (first layer of septins on membranes) and perpendicular to the 1D wavy line of the substrate (second layer of septins bound to the first layer of septins), respectively. Measurements were performed depending on the concentration of septins in solution (*d*_1_=46±20 nm and *d*_2_=55±24 nm in 26 nM, *d*_1_=27±11 nm and *d*_2_=19±8 nm in 87 nM). Bars show the mean±s.d. Images are representative of four experiments.

**Fig. 3.**
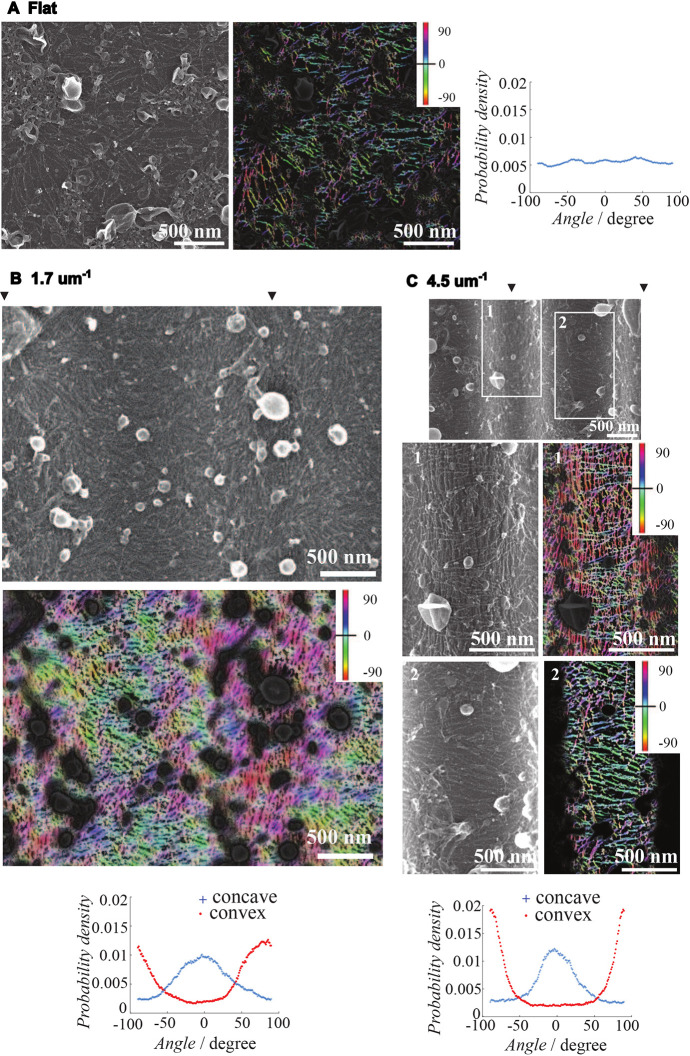
**SEM of septin filaments on lipid bilayers supported by undulated solid substrates of curvatures ranging from 0 to 4.5** **µm^−1^**. SEM images of septin filaments at 26 nM concentration incubated with flat and varying curvatures of undulated solid substrates. (A) Raw SEM image of septin filaments on a flat NOA71 substrate (left). Segmented filaments colored according to their orientations were overlaid on the raw SEM image and are shown in the middle panel. The distribution of filament orientations averaged from three positions is plotted in the graph on the right. (B) Raw SEM image of septin filaments on an undulated substrate with curvature ranging from −1.7 to +1.7 µm^−1^ (2.1 µm periodicity, 0.20 µm amplitude) (top). (C) Raw SEM images of septin filaments on an undulated substrate with curvature ranging from −4.5 to +4.5 µm^−1^ (1.9 µm periodicity, 0.45 µm amplitude). An image covering a wide range is shown on top, and images focused on the boxed regions 1 (convex) and 2 (concave) are shown in the middle panels. For B,C, segmented filaments colored according to their orientations were overlaid on the raw SEM images and are shown in the middle (B) or middle right (C) panels. The distributions of filament orientations at concave and convex parts averaged from three positions for each part are plotted in the graphs on the bottom. Black arrowheads show the tops of convex hills of the substrate. Images are representative of two sets of independent experiments.

Budding yeast septins are known to be sensitive to micrometric curvatures (Beber et al., 2019b; Cannon et al., 2019), as opposed to most curvature-sensitive proteins (Mim and Unger, 2012). We thus tested whether human septins could sense curvature at the micrometer scale as well. To this end, septin filaments were incubated with supported lipid bilayers, deposited on undulated wavy solid substrates designed to display a micrometer periodicity. Both the amplitude and the periodicity of the substrates can be finely tuned (Nania et al., 2015, 2017). We tested wavy substrates with peak curvatures of 1.7 µm^−1^, 3 µm^−1^ and 4.5 µm^−1^. These parameters were set after previous reports suggested a micrometer curvature sensitivity of octameric budding yeast septins (Beber et al., 2019b; Cannon et al., 2019). Also, we highlighted vesicle deformations showing this curvature range (Beber et al., 2019b). The resulting samples were imaged by scanning electron microscopy (SEM) (Figs 2 and 3). Lipid bilayers strongly interact with the substrates and are thus not deformable. The wavy substrates from Fig. 2 displayed a periodicity of 1.6 µm and an amplitude (from peak to peak) of 200 nm, corresponding to curvatures ranging from −3 to +3 µm^−1^ (Nania et al., 2017) (see Materials and Methods). Solutions of recombinant human septin octamers arranged as: SEPT2–GFP-SEPT6-SEPT7-SEPT9_i1-SEPT9_i1-SEPT7-SEPT6-SEPT2–GFP (concentrations ranging from 8 to 87 nM) were incubated with the substrates covered with a lipid bilayer, in a 75 mM NaCl, 10 mM Tris pH 7.8 buffer. The molar septin concentrations are concentrations of the octameric complex rather than the concentration of individual septin subunits. Within all of the SEM images, additional vesicles could be spotted bound to samples even though thorough rinsing was performed. Indeed, free PI(4,5)P_2_-containing vesicles in solution might interact strongly with septins and thereby still decorate filaments. At low protein concentrations (8 nM), barely any septin filaments were detected within the convex sections of the substrates, whereas polymerized septin filaments were observed at concave sections (Fig. 2A). Single septin filaments thereby presented a higher affinity for concave (negative) curvatures than for convex (positive) curvatures. Interestingly, the orientation of septin filaments in the concave region was specific. Indeed, septin filaments were oriented perpendicular to the longitudinal axis of 1D periodic substrate waves. At 26 nM septin concentrations (Fig. 2B), septin filaments were still present at the concave negative curvature of the substrate. Additionally, they were distributed at convex (positive) curvatures as well. The orientation of the filaments in the convex (positive-curvature) sections was orthogonal to the orientation of the filaments present on the concave (negative-curvature) sections. Hence, on convex regions, the filaments remained straight and unbent, following the direction of the periodic wavy lines of the substrate (Fig. 2B, middle). In addition, in the same concentration range (26 nM bulk septin concentrations), local higher septin densities displaying an alternative organization were observed. This observation suggests that 26 nM was close to a threshold bulk septin concentration at which a drastic re-organization of septins was visualized (Fig. 2C; Table S1). Indeed, septin filaments from the concave domains extended towards convex domains and were superimposed orthogonally to the straight septin filaments running along the convex hills, forming mesh-like structures over two layers (Fig. 2C). This mesh-like organization reflected similar conclusions obtained from cryo-EM (Fig. 1). Distances between adjacent septin filaments (*d*_1_=46±20 nm and *d*_2_=55±24 nm; Fig. 2E) in the mesh-like organization were distributed around approximatively 50 nm. In addition to this specific mesh organization, another filamentous pattern was detected. Filaments from concave domains were merged with and seen running alongside adjacent filaments on convex domains by assembling into semicircular patterns (diameters of about 0.8 µm) (Fig. 2C; Fig. S3A). Above 44 nM septin concentrations (Fig. S3B), both convex and concave regions of membranes were already fully covered with a meshed network of septins. To ensure full saturation of the surface with septins, we used a higher concentration of 87 nM (Fig. 2D). Interestingly, we could not detect any intermediate between the sparse filamentous decoration observed at 26 nM and that observed at the maximal saturation with proteins. At full saturation, the mesh-like organization in two layers was ubiquitous and thus present at both concave and convex domains (negative and positive curvature, respectively; Fig. 2D), resulting in more regular periodicities in distances between filaments (*d*_1_=27±11 nm and *d*_2_=19±8 nm; Fig. 2E) (see signals around 1/30 nm^−1^ in 2D Fourier transformation in Fig. S3C). The typical spacing recovered from those dense meshes, on average, reflected the octameric periodicities of human septins (32 nm). Besides, we never observed, even at the highest protein concentrations tested (up to 87 nM), any additional third protein layer on top of the described orthogonal mesh, suggesting that all available septin–septin contacts must be implicated in building the two-layer mesh, even at high septin concentrations.

In Fig. 3, images from alternative (shallower and sharper) as well as flat curvatures for initial septin concentrations of 26 nM are shown. At this concentration, septins reached a maximal protein density (Fig. 2B,C). Bound to flat membranes, septin filaments organized in random orientations even though, within domains, they could still be parallel to one another (Fig. 3A), reflecting their organization into a nematic-like pattern. The orientation of the filaments was analyzed for all tested curvatures. Fig. 3A (right panel), Fig. 3B (lower panel) and Fig. 3C (lower panel) recapitulate the preferential orientations. Except for the distributions seen on the flat surface, the resulting angular distributions were similar, and septins, on average, lay straight on the positively curved areas of the ‘waves’ and bent negatively within the negatively curved surface. Bounded to shallow curvature (1.7 µm^−1^), we also noticed that the septin filaments were organized within domains of about 500 nm in diameter. The spatial extent to which filaments were oriented similarly was significantly smaller (about 500 nm) than for higher curvatures (several micrometers). This suggests that substrates of higher curvatures impose the orientation of septin filaments at a larger scale, imposing a stronger obstacle for septins to orient randomly.

### Coarse-grained model to capture the organization of septins on wavy substrates

**Fig. 4.**
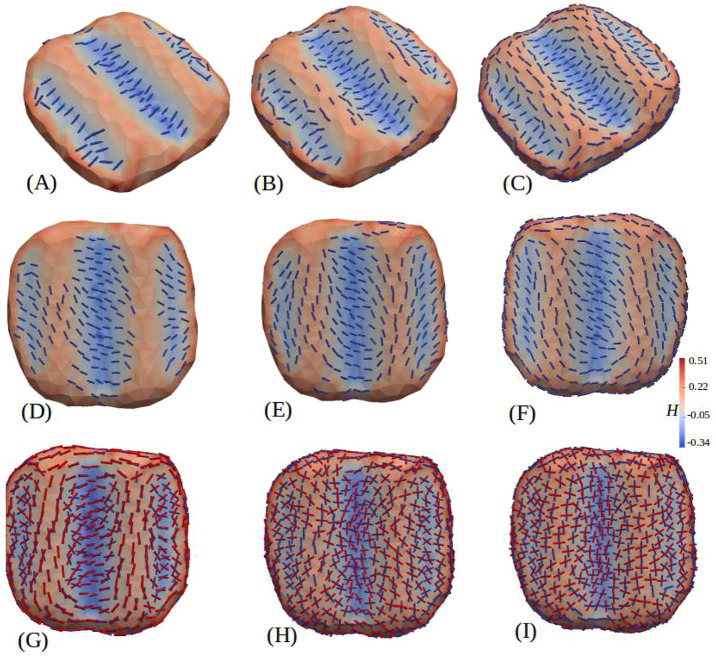
**Simulation on a wavy patterned surface.** Monte Carlo simulation of our model of septin on a rigid wavy substrate, with the intrinsic curvature of septin being 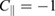. The color bar represents the local mean curvature of the substrate. Panels A–C have one layer of septin, whereas panels G–I have two layers of septin. Reduction of the intrinsic curvature (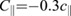) (D–F) changes the alignment of septins in the valleys (blue) towards the larger radius of curvature, oblique to the direction of undulation. The septins on the hills (red) also turn and form circular structures along with the ones in the valley (F). In A–C and D–F, the amount of septins was increased gradually from left to right. In G–I, the strength of interaction (*ε*) between the two layers of septin was increased from left to right (*ε*=1, 3, 5 and 6). In I, with *ε*=6, the orientations in the two layers became fully perpendicular. The other parameters were *κ*=20, 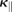
=25, *κ*_⊥_=25 and *ε*_*LL*_=2 in *k_b_T* units, and *C*_⊥_=0 in arbitrary units. All parameters were the same for both layers of proteins.

We employed a phenomenological model that had been used earlier to explain how nematic filaments can organize themselves on deformable membranes to reshape them (Kumar et al., 2019; Ramakrishnan et al., 2013). The model is based on the ability of filaments to deform the underlying membrane anisotropically and the tendency of these filaments to arrange parallel to each other, forming nematic order. This model had been used for various systems such as GUVs coated with FtsZ filaments having intrinsic curvatures, as well as GUVs coated with DNA molecules displaying no intrinsic curvature (Franquelim et al., 2021; Kumar et al., 2019). Both systems showed the formation of membrane tubes. So far, this model has been used to describe the behavior of a single layer of filaments arranged on a deformable membrane (Kumar et al., 2019; Ramakrishnan et al., 2013). In the present work, we have adapted this model to a multilayered system in order to describe how two layers of septin filaments can arrange perpendicular to each other when bound to a non-deformable membrane. We thus modeled the septin-coated membrane as two nematic fields adhering to a fluid membrane surface. The first one directly adheres onto the membrane, whereas the second one interacts with both the membrane and the first layer of protein. The details of the model are presented in the Materials and Methods. The main results of this model, obtained by Monte Carlo simulations, describe the arrangement of septins on non-deformable wavy surfaces, similar to our wavy curved experimental substrates (Fig. 4), and are qualitatively consistent with the experimental results. The simulation results in the presence of a single septin layer are presented in Fig. 4A–F. At low septin density, filaments first populate the valleys, orientating themselves along the curved concave surface with negative curvature, similar to the intrinsic curvature of the filament itself, i.e. 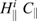 (Fig. 4A), where 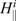 is the local curvature of the surface along the filament length and 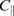 is the intrinsic curvature of the filament. As both 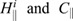 are negative here, the energy cost 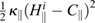 is minimized, where 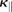 is the induced membrane bending rigidity. Furthermore, at a higher density, filaments distribute onto the convex part of the wavy surface (the crests) but orient parallel to the channels along the non-curved direction with zero curvature (Fig. 4B–F), i.e. 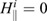, in agreement with Beber et al. (2019b). The corresponding energy cost 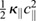 is the minimum, where 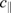 is the induced intrinsic curvature, because for any other orientation of the filament on this convex cylindrical shaped surface, 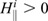, and thus 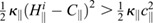. In case the intrinsic curvature of septins was lower than that of the troughs of the wavy substrate, the filaments made an angle with the transverse direction to the channel (Fig. 4D–F), seeking out lower 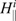 to match with their own intrinsic curvature. This was also pinpointed in our experimental results in which filaments were slightly tilted sideways from a pure orthogonal orientation to the wavy undulations (Fig. 2A,B). At higher septin densities, simulations (Fig. 4F) presented circular patterns spanning the convex and concave parts of the channels, which were similar to the experimental observations (Fig. 2C; Fig. S3A). Fig. 4G–I shows the effect of an additional second layer. In our model, two filaments at the same location but belonging to two different layers interacted via a repulsive interaction strength *ε*, which favored a mutually orthogonal orientation between the two filaments (see the ‘Model for Monte Carlo simulations’ section in the Materials and Methods). From left to right in Fig. 4G–I, *ε* was increased. Note that here, the intrinsic curvatures of filaments did not change in both the first and second layers. For low *ε* (Fig. 4G), both layers tended to align parallel to one another in the channel troughs and crests, even at the cost of *ε*. However, when *ε* dominated over the membrane–septin interaction, both layers could not simultaneously minimize their interaction energy with the membrane. At relatively high interaction strength (*ε*), the filaments were orthogonal to each other (Fig. 4I) across the layers.

### Micrometric membrane reshaping by polymerization of human septin octamers

**Fig. 5.**
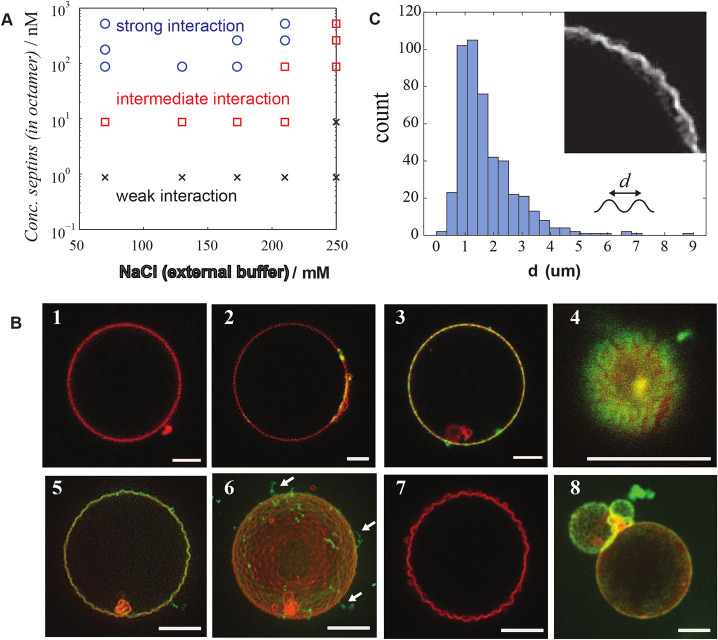
**Characterization of the interaction between human septins and membrane.** (A) Phase diagram of GUV behavior as a function of concentration of NaCl in external buffer and concentration of human septin octamers. (B) Confocal fluorescence microscopy images of slices of the GUVs. Lipids and septins are visualized in red and green, respectively, and these signals are overlaid. (1) A GUV without human septin octamers in 10 mM Tris buffer at pH 7.8 containing 70 mM NaCl. (2) An example image of a GUV found in the region ‘weak interaction’. The GUV was incubated with 0.87 nM septin in 10 mM Tris buffer containing 70 mM NaCl. (3,4) Examples of GUVs partially covered by septins. GUVs were incubated with 4.4 nM septins in 10 mM Tris buffer containing 70 mM NaCl. Image 4 is a 2D image and recorded at the bottom of the surface of a GUV. (5,6) Deformed GUVs found in the region ‘strong interaction’ in the phase diagram. 2D- and 3D-reconstituted images of GUVs with 176 nM septins in 10 mM Tris buffer containing 70 mM NaCl. White arrows indicate excess of septins polymerized in solution. (7) A deformed GUV with 36 nM of dark septins in 10 mM Tris buffer containing 70 mM NaCl. (8) Ring-like organization of septins bound to membranes at high-salt condition. An image of 3D-reconstituted GUV interacting with 520 nM septins in 10 mM Tris containing 250 mM NaCl. Scale bars: 5 µm. Images are representative of four experiments. (C) Distance between peaks of bumps in deformed GUVs (*n*=18 vesicles). Different concentrations of septins are plotted on the graph. Each gives a similar distribution (see Fig. S5A). The image shows detail of a deformed GUV. Scale bar: 5 µm.

**Fig. 6.**
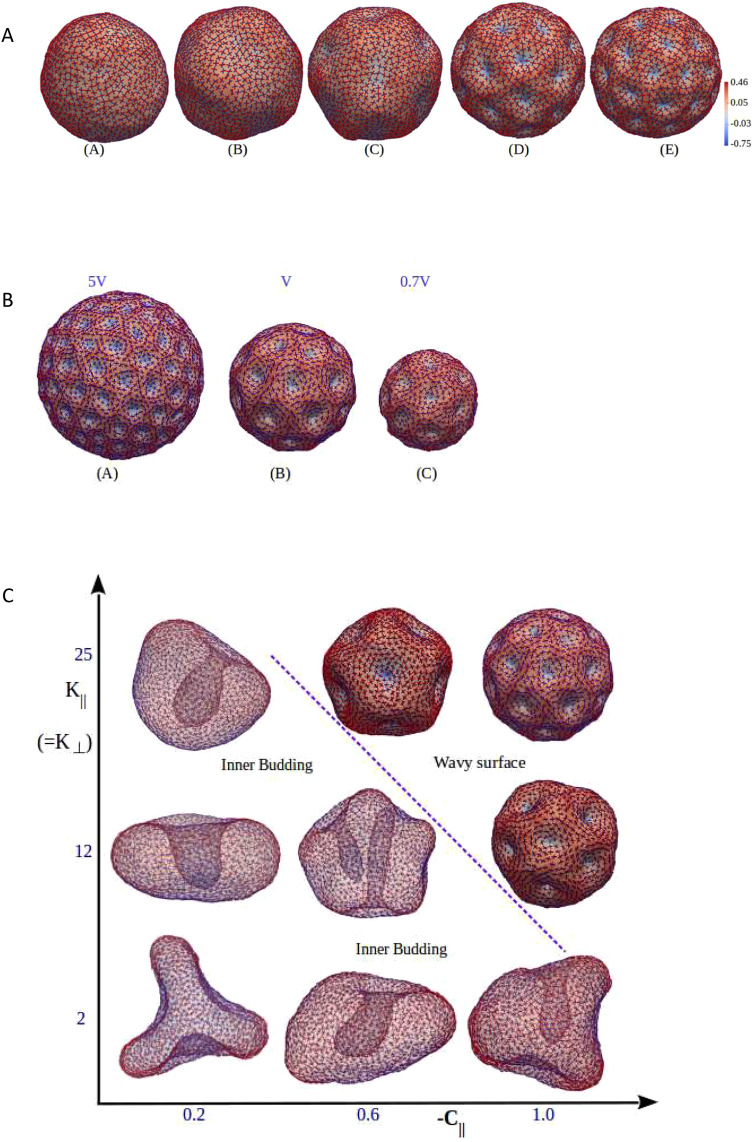
**Simulation describing membrane deformations by nematically ordered filaments.** (A) Simulation of septin on a deformable vesicle. Interaction between the second layer and the membrane is reduced gradually from model A to model E. The observed wavy surface emerges when only the first layer interacts with the membrane (models D and E). The ratio of the corresponding induced bending rigidities for both layers 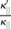 and 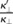 are 1 (in model A), 0.5 (in model B), 0.1 (in model C), and 0 (in models D and E). *κ*_||_ and *κ*_⊥_ values are 5, 10, 15, 25 and 40 for models 1–5, respectively. The other parameters are *κ*=20, *ε*_*LL*_=1 and *ε*=1 (in *k_b_T* units), 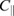
=−1 and *C*_⊥_=0. All parameters are the same for both layers of proteins. The color bar indicates the local mean curvature of the surface. Note that number of dips saturate at higher *κ*_||_. (B) Surface morphologies are shown from simulations performed at different vesicle volumes, keeping all other parameters fixed, corresponding to the configuration 4 shown in A. This shows that the distances between the surface dips remain approximately the same for different-sized vesicles. (C) Phase diagram of vesicle shapes as a function of anisotropic bending modulus *κ*_||_ and intrinsic curvature 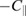. The experimentally observed undulated GUV shapes emerge when both these parameters are high, whereas handful of buds and tubes occur when both these parameters are small (see detailed explanation in the main text). The dotted line indicates the threshold between the observed experimental deformations and simulated deformations.

**Fig. 7.**
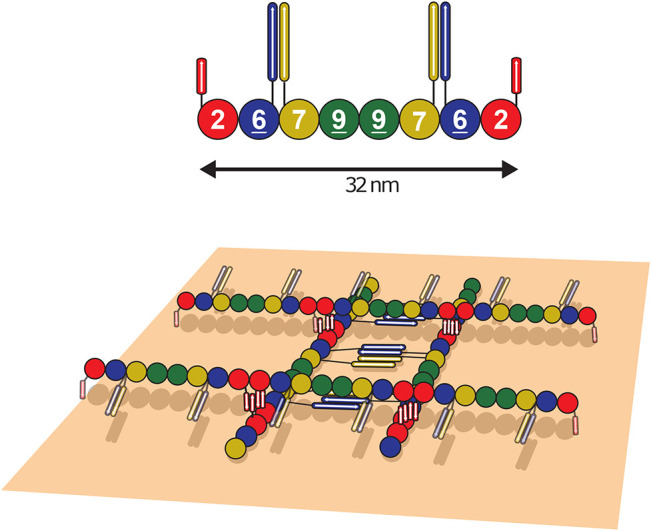
**Model of orthogonal network assembly.** Schematic of octameric septins (top) and a possible model of the orthogonal network array of human septin filaments. Note that the orientation of parallel coiled coils in the second (upper) layer of septins could diverge from this proposal in terms of orientation.

Given that septin filaments are arranged in a specific manner on micrometric membrane curvatures (Figs 2 and 3), one could assume that septin filaments could reshape free deformable membranes. We thereby examined how septins could deform and reshape the membranes of GUVs by confocal fluorescence microscopy. To finely describe how septins deform membranes, a phase diagram was built in which both the protein and salt concentrations were varied. GUVs containing both DOPS and PI(4,5)P_2_ were prepared and osmolarities within GUVs were carefully adjusted to match the external buffer osmolarity in order to avoid any osmotic shock. Fig. 5 presents a typical observation following the incubation of GUVs with human GFP–septin octamers for more than 1 h at room temperature. Without septins, the control GUVs exhibited a rather smooth membrane (Fig. 5B-1). At low bulk septin concentrations (about 1 nM), septins did not distribute homogeneously on membranes (Fig. 5B-2), represented as ‘weak interaction’ in Fig. 5A. Instead, septins localized at minor membrane defects (small vesicles or lipid aggregates attached on the surface of GUVs) or at vesicular inter-connections (Fig. S4A). When increasing the septin concentration, the surface coverage of septins on GUVs rose (Fig. 5B-3). In ionic strength conditions below 175 mM NaCl, micrometric deformations of GUVs were observed in more than 30% of vesicles, above 10 nM septin concentrations (‘intermediate interaction’ in Fig. 5A). The deformation rate increased with increasing septin concentrations. At septin concentrations above 100 nM (‘strong interaction’ in Fig. 5A), more than 50% of the observed vesicles were deformed. Reshaping thus required a sufficiently high density of septin filaments on membranes. Most of the deformed GUVs displayed micrometric periodic ‘bumpy’ convex shapes, which remained static when imaged for about 1 min. The observed deformations were often irregular (Fig. S4B) (∼70%, *n*=62 vesicles from six experiments), whereas some ‘bumps’ (∼30%) were regularly distributed and similar in dimensions (Fig. 5B-5,B-6). The membrane surface was thus covered with convex periodic bumps (enlarged in Fig. 5C). In addition, the periodicity of these bumps was independent of the protein density (Fig. S5A). We checked that these deformations did not result from the presence of GFP covalently attached to septins. Indeed, bumpy GUVs were also observed using dark human septin octamers (Fig. 5B-7). In high-salt buffer (above 250 mM NaCl, conditions in which the polymerization of septins is inhibited), at any septin concentration, most GUVs did not undergo membrane reshaping, even though GUVs were fully coated with septins. Therefore, membrane reshaping is correlated with the polymerization of septin octamers into filaments. Besides, we often visualized filamentous protein structures protruding outwards from GUVs (Fig. 5B-6, arrows). Those protrusions only contained septins as green fluorescence could exclusively be visualized at those locations. Because septins cannot accumulate into multiple layers on GUVs, the excess protein protruded as filaments or, most likely, bundles of filaments. The observed bumpy deformations in deformed vesicles displayed around a 2 µm periodicity [1.8±1.1 µm (mean±s.d.), median=1.4 µm, *n*=18 vesicles] (Fig. 5C), which is in agreement with the periodicity of membrane deformations induced by yeast septin octamers (varying from 2 to 6 µm) (Beber et al., 2019b). However, the curvature of the deformations (convex in these cases) was the opposite of that imposed by budding yeast septins (concave). In addition, analysis of deformations from vesicles displaying a range of radii (3–12 µm) showed that the periodicity of local deformations barely varied (Fig. S5B).

In non-polymerizing conditions (>250 mM monovalent salt concentration) and at high septin concentration (>260 mM), septins organized into rings. Their diameters were measured at 0.96±0.13 µm (*n*=11 vesicles from three experiments) (Fig. 5B-8). At these ionic strengths, electrostatic attractive interactions between positively charged septins and negatively charged lipids should be screened. Without PI(4,5)P_2_ and in the presence of charged DOPS only, those rings did not self-assemble anymore (Fig. S4C,D). The requirement of PI(4,5)P_2_ at high salt concentrations suggested that the septin–membrane interaction in high-salt conditions was driven by non-electrostatic septin–PI(4,5)P_2_ interactions.

We have demonstrated here that human septin complexes deform GUVs in a periodic fashion, with periodicities similar to those imposed by our engineered wavy patterns (Fig. 2). The observed mesh-type structure of septins on supported lipid bilayers at high septin concentration probably reflects the septin organization on deformed giant vesicles. The obtained periodicities in bumpy deformations in GUVs (about 2 µm) were similar to the periodicity existing in 1D wavy substrates (1.6 µm). Interestingly, filaments at the concave region of the supported lipid bilayer are not oriented perfectly perpendicular to the longitudinal axis of the 1D periodic wave of substrates and appear to relax their bindings by tilting from the perfect perpendicular angles.

To better interpret our observations, we used the coarse-grained model that was used previously to describe septin orientation on wavy non-deformable substrates (Fig. 4). However, in this case, the septins resided on the deformable fluid membrane of a GUV (see Materials and Methods). Therefore, the filament–membrane interaction could, in this context, tune the membrane and/or vesicle shape. We also considered the same repulsive interaction *ε* that promotes the local orthogonal orientation between septins that sit on both layers. Fig. 6 shows the results obtained for a fluid, deformable surface holding two layers of nematically organized filaments. Experimental observations showed that membrane deformations frequently occur when septin concentrations are high (Fig. 5). Septins were observed to organize themselves in two layers. In the experimental results, the second layer appeared before full saturation of the first layer. This implies that above a given concentration, septin deposition on the vesicle increases on both the first and second layers because of the increase of septin concentration in the solution. We assumed that the increase in septin density on the first layer weakened the septin–membrane interaction of the second layer, because the membrane surface was dominantly covered with the septins from the first layer. Hence, at high concentrations, the effect of the second layer became weaker, whereas the effect of the first layer became stronger. We implemented this in the simulation by increasing 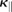 and *κ*_⊥_ of the first layer and simultaneously decreasing 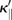 and 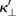 of the second layer with increasing septin concentrations. Based on these hypotheses, the deformation of GUVs could be reproduced in our simulation (Fig. 6). At low concentration, both layers had equal strengths of interaction with the membrane and our simulation showed that for 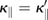, all undulations vanished, resulting in a smooth spherical surface. When 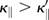 and 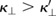 (Fig. 6AC–AE), membrane deformations similar to our experimental observations were obtained. Membrane deformations can thus be dominated by the interaction between the first layer of septin and the membrane. At low concentrations of septin, when 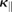 and 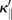 were estimated to be equal, the membrane-deforming ability of the two layers canceled each other, provided that 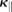 and 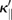 were small in magnitude relative to *ε*, ensuring locally orthogonal alignment of septins in the two layers. This was a non-trivial effect because, at a given point on the membrane, one could consider an isotropic concave valley (i.e. part of an inverted hemi-spherical bowl), which could accommodate two septin filaments that were mutually orthogonal and belonging to the different layers. However, in a volume-preserving scenario (which corresponded to our conditions), valleys would also be accompanied by hills for which the surface curvature was positive and, therefore, unfavorable to filaments (as 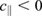), thus causing a positive unfavorable energy for septin membrane interaction. In the simulation, we note that this is indeed the case when the intrinsic curvatures of filaments are identical in the first and second layers. In a realistic situation, septins can be oriented differently at the first and second layers, which can change the effect of the second layer on membrane deformations. In addition, surface tension also opposes surface undulations. It would appear that instead of forming these valleys and hills, the vesicle prefers a smooth surface, which has a low positive energy cost. An approximate energy comparison is given in the Materials and Methods. In our simulations, we also varied the dimensions of the vesicle to show that the periodicity of the local bumpy deformations does not vary with GUV diameter (Fig. 6B). The simulation thus confirms that the periodicity of the deformations does not depend on the vesicle dimensions (Fig. S5B).

In Fig. 6C, we present an approximate phase diagram of deformed GUV shapes as a function of *κ*_||_ (bending modulus) and 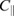 (intrinsic curvature of septins). At relatively high 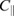, many small dips form, whereas at smaller 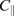, the dips become wide and thus fewer dips appear. Note that the formation of buds or tubes require extra membrane area, which is restricted by surface tension. Therefore, when the number of buds and/or tubes remains low, they can grow without competing with others. In contrast, at higher 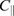, competition among the large numbers of dips inhibit individual dips to grow as inner tubes. Additionally, the nucleation of buds and/or tubes is energetically easy for low *κ*_||_. Note that the inner tubes in this case are not formed due to bundling of filaments, but due to 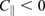 and low *κ*_||_ (Kumar et al., 2019). Our experimental observations thus correspond to relatively high *κ*_||_ and 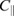. Given that 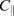 is an intrinsic property of septins, it cannot be tuned. Even using GUVs with membranes of low bending modulus, such inwards invaginations were not observed. Previously, outwards tubulations were reported (Tanaka-Takiguchi et al., 2009) from GUVs induced by the human hexameric septin complex (SEPT2-SEPT6-SEPT7). However, in our conditions, we did not observe any tubulations.

Our study thus highlights the specific behavior of the human octameric septin complex on membrane interaction, reshaping and curvature sensitivity, indicating that these properties are conserved between species.

## DISCUSSION

After investigating the behavior of budding yeast septins bound to the membrane, clues from observations in solution (Iv et al., 2021; Szuba et al., 2021) and septin localization *in vivo* (Lindsey and Momany, 2006) indicated that metazoan septin interaction with lipids would have unexpected and novel outcomes, as compared with those observed for fungal septins. In solution, human septin octameric complexes self-assemble, similarly to *Drosophila* septins (Mavrakis et al., 2014). These metazoan octameric septin complexes spontaneously bundle through lateral filamentous interactions (Bertin et al., 2008; Garcia et al., 2011; Iv et al., 2021; Kinoshita, 2003; Mavrakis et al., 2014; Szuba et al., 2021), and appear as straight spine-like or circular bundles of paired or single filaments (Fig. S6). On membranes, *Drosophila* septins or hexameric human septins form tight sheets of parallel filaments (Szuba et al., 2021), similarly to budding yeast septins. On the contrary, thoroughly studied budding yeast septins assemble in cell-free assays into stable paired filaments and only occasionally bundle (Bertin et al., 2008; Garcia et al., 2011). Budding yeast and human septins thus display different behaviors of self-assembly. Even though septins have evolved from an ancient ancestor (Shuman and Momany, 2022), phylogenetic analyses have shown that within the seven subgroups in which septins are classified, only one group (1A) encloses both budding yeast Cdc10 and human SEPT9. Besides, human septins and, in particular, SEPT9 can be expressed as a variety of splice variants, which contrasts with fungal septins (Hall and Russell, 2004). Also *in situ*, electron tomography has shown that septins would at least transiently be able to organize into a mesh-like structure at the division furrow (Bertin et al., 2012). Additional partners might thereby promote extra protein–protein contacts *in vivo* to generate an orthogonal mesh.

We therefore used a combination of methods to follow the interaction and curvature preference of human septins bound to liposomes at different scales (ranging from a few tens of nanometers to micrometers). We analyzed the organization of the human septin octameric complex interacting with wavy substrates of varying micrometer curvatures and coated with supported lipid bilayers. Using electron and fluorescence microscopy, we were able to describe the features of our samples at different scales (from nanometer to micrometer ranges).

In the presence of a biomimetic membrane (supported lipid bilayer or monolayer), we propose that the septin–membrane interaction prevails over the septin–septin lateral association, preventing the self-association of thick bundles. Single or paired filaments thus assemble onto membranes without bundling. We found that human septin octamers systematically assemble into arrays of orthogonal filaments, contrary what is seen for budding yeast, *Drosophila* or human hexameric septins, which preferentially self-assemble on membranes as parallel filamentous arrays and only occasionally arrange as networks of filaments (Beber et al., 2019b; Bertin et al., 2010). It has recently been shown *in vivo* that septins are close to the membrane, acting as an intermediate to connect actin to lipids (Martins et al., 2023). Moreover, orthogonal gauzes of human septin filaments were consistently observed on liposomes (Fig. 1) or on supported lipid bilayers (Fig. 2C,D; Fig. S3B). The inter-filament distance (around 30 nm) on an undulated substrate, extracted from SEM images, agrees either with the spacing of paired filaments or with an octameric periodicity. At the highest densities, the first layer of septin filaments directly apposed onto the lipids displays a regular spacing of about 30 nm, in agreement with a septin octameric length. The second septin layer sits orthogonal to the first layer. Within the second layer, our analysis suggested that filaments are closer to one another (10–20 nm), which would coincide with filament pairing mediated by coiled coils. To induce this network, the first septin layer would need to be specifically arranged in a parallel fashion and create a scaffold to recruit the second perpendicular layer.

Based on the molecular specificities of septins, Fig. 7 displays a molecular model that would account for our observations. X-ray crystallography studies (de Almeida Marques et al., 2012) suggest the versatility of the possible septin–septin interactions mediated through the coiled coil domains of SEPT6, SEPT7 and SEPT2. Antiparallel or parallel arrangements of SEPT2–SEPT2 coiled coils as well as homodimeric or heterodimeric, parallel or antiparallel coiled coils involving SEPT6 and SEPT7 can be generated. In our samples, the lateral organization between adjacent filaments in the first layer is probably triggered by the pairing of filaments mediated by coiled coils (most likely antiparallel coiled coils involving SEPT6–SEPT7; Leonardo et al., 2021). The filament side interacting directly with the membrane could mediate its interaction through the α0 domain (PB1) of septins. Leonardo et al. (2021) suggest that the space between the coiled coil and the membrane could integrate a chain of hydrogen bonds because of the limited rotation of the coiled coil superhelix, thus displaying a hydrophilic side towards the membrane. The septin–membrane interaction would thus be stabilized through the coiled coils. The presence of polybasic regions at the very end of SEPT6 and SEPT7 C-termini also strengthen the septin–membrane interaction. The first septin layer should, in turn, interact with the second layer, possibly from SEPT2–SEPT2 antiparallel coiled coil interactions, one helix from each layer (Fig. 7). The second layer would thus stabilize the first septin layer and induce an octameric repeat between the septin filaments from the first layer. The higher density of the second septin layer might impose a higher ordering of the first layer. Short distances (10–20 nm) are indeed observed between filaments in the second layer. It is possible that two filaments are closely paired and juxtaposed when interacting with the first septin layer or connected via coiled coils. Besides, we believe that coiled coil pairing remains rather flexible and induces both the measured dispersity in the inter-filament distances and enough flexibility to allow, to some extent, adaption of the septin filaments to curvatures. Finally, only two layers of filaments can be observed. Septins, thus, do not accumulate into successive layers, suggesting that the coiled coils that possibly mediate the interaction are already engaged in the septin–membrane and septin–septin interactions. A similar behavior had previously been observed for *Drosophila* septin organization onto membranes (Szuba et al., 2021).

The curvature sensitivity of septins at the micrometer scale has been first highlighted in fungi *in vitro* and has been discovered and intensively investigated in the budding yeast model (Beber et al., 2019b; Cannon et al., 2017, 2019). Given that human septins organize in a more complex network, this model needs to be revisited. In mammalian cells, septins also interact with membranes at specific locations at which both positive and negative micrometric curvatures are present (Hu et al., 2010; Karasmanis et al., 2019; Shen et al., 2017). The specificity of septins to micrometric membrane curvatures is probably firmly inherited over species. As septins are involved in membrane remodeling processes, particularly human septins, revealing the mechanisms of membrane curvature sensitivity at the molecular level is key to understanding the mechanisms of cellular functions involving human septins. We suspect that the curvature preference might result from the intrinsic curvature of the septin rods. Indeed, the Garratt laboratory showed that human septin hexamers were bent, with a deflection of 22 Å from the central axis (Mendonça et al., 2021).

From the discrepancies observed between human and yeast septin ultrastructural organization, it is thus not surprising to report distinct behaviors for membrane reshaping and curvature sensitivity. In budding yeast, a dense parallel set of septin filaments deforms GUVs *in vitro* to generate negative micrometric curvatures. These concave deformations and the correlated curvature sensitivity imposed by budding yeast septins were modelled using an enhanced membrane affinity energy with the filaments bent negatively (Beber et al., 2019b). For human septins, the behavior, in terms of curvature sensitivity, is similar to that of budding yeast septins at low concentrations. At low septin concentrations, at which the filament density is not sufficient to induce membrane deformations, human septins do not assemble into orthogonal arrays. Instead, they organize as individual filaments (Fig. 2A,B) and bend negatively on concave geometries, whereas they remain unbent when encountering a hilly positively curved domain. Conversely, at higher protein concentrations, orthogonal gauzes can be visualized on both positively and negatively curved domains with similar densities. Our Monte Carlo simulations, which consider septins as nematically ordered filaments, closely reproduce these experimental observations. The transition from a low filament density to a dense network of filaments occurs within a short range of concentrations (30–40 nM). We were unable to detect any intermediate state in terms of protein density on supported lipid bilayers, suggesting that a cooperative effect tends to facilitate the formation of networks. This network-like organization most likely reflects the organization of human septins when bound to GUVs. Indeed, on GUVs, the observed deformations follow a curvature, the dimensions of which correspond to those of our wavy substrates. A dense orthogonal network of human septins thus generates positively curved deformations, as also reflected by the simulations (Fig. 6). We can thus assume that the formation of an orthogonal network of filaments is key to bolster the emergence of membrane deformations in the scope of our current report.

Tanaka-Takiguchi et al. (2009) previously captured outwards tubulation events (i.e. concave deformations) on GUVs incubated with either brain extract or hexameric human septin complexes (SEPT2-SEPT6-SEPT7). Our simulation suggests that at both low bending modulus and septin intrinsic curvature, inwards tubulations are induced instead of outwards tubulation. This discrepancy from the behavior visualized by Tanaka-Takiguchi et al. (2009) might result from the nature of the septin complex considered. Indeed, outwards tubulations were seen for hexameric human septins, which have a tendency to organize onto parallel sheets of filaments, instead of the orthogonal arrays obtained with septin human octamers. Experimentally, hexameric septins were organized circumferentially, perpendicular to the tube axis. Such micrometric GUV deformations induced by the collective behavior of nematic-ordered filaments has been also discussed in other reports (Kumar et al., 2019; Ramakrishnan et al., 2013). For example, similar GUV deformations, where GUVs can be deformed and wrinkled before tubulation occurs, were observed using DNA origami constructs capable of self-assembling into filamentous structures (Franquelim et al., 2021). Franquelim et al. thereby propose that the polymerization of filaments bound to membranes can control membrane reshaping. Our Monte-Carlo simulation (Fig. 6) has shown that the negative intrinsic curvature of septin (i.e. concave) is able to produce both convex hills and concave valleys on GUV surfaces. The first layer of septin (blue in Figs 4 and 6), which is dominant, orients on the valleys in a vortex-like pattern, exploiting the concave shape of the valley. On the convex parts of the vesicle, between two valleys, septin filaments orient along the direction that has minimal positive curvature. This is identical to their orientation on the wavy substrate. The second layer of septin (red in Figs 4 and 6), which has a relatively weak effect on vesicle deformation, orients along the downhill concave direction towards the bottom of the valley, satisfying its own negative intrinsic curvature. We found that the valleys have the shape of inverted hemispheres rather than pointed inverted cones with reducing apex angle. This energetically benefits both the layers of septin as they attain preferred negative curvature. On the convex hills, both layers pay the energetic price.

Kumar et al. (2019, 2022) have pointed out from their Monte Carlo simulations that the intrinsic curvatures of filaments imposing anisotropic membrane curvatures (both longitudinal and orthogonal to the direction of the filaments) can drive either concave or convex deformations of liposomes, with the filaments ordered in a nematic fashion. We imposed negative intrinsic curvature for human septin filaments along their length because in yeast, septin filaments were also found to have negative intrinsic curvature of similar magnitude. However, yeast septins showed additional bundling interactions, which are absent for human septins. This general model of nematic filaments interacting with GUVs, imposing anisotropic membrane curvature, has been relevant for other systems as well. For example, artificially tuning the intrinsic curvature of FtsZ can trigger the emergence of either concave or convex deformations and tubulations on vesicles (Osawa et al., 2009). However, membrane curvature and vesicle tubulation can also be induced by chiral nematics having no intrinsic curvature or bundling interaction (Behera et al., 2021). Overall, application of this model to human septins allows us to rationalize our experimental observations of the orientation pattern of septins on rigid wavy surfaces as well the nature of deformations of vesicles.

### Conclusion

We studied, *in vitro*, the organization of human septin octamers (SEPT2–GFP, SEPT6, SEPT7 and SEPT9i1) on reconstituted model lipid membranes. Using electron microscopy, we revealed that human septin filaments assemble into a perpendicular mesh-like network on membranes constituted with two layers of nematically ordered septin filaments perpendicularly bound to each other. Human septins were able to reshape soft deformable membranes (GUVs). On an undulated supported lipid bilayer with micrometric periodicities similar to the scale of these deformations, the filaments oriented according to the local curvatures, resulting in a regular mesh-like structure in terms of the distances and angles. Compared with septins from other species (yeast and fly), human septins organize in a more complex network. Our Monte Carlo simulations could recapitulate the behavior of human septins, considering the filaments as rod-like nematic liquid crystalline objects.

## MATERIALS AND METHODS

### Chemicals

Common reagents (ethanol, acetone, chloroform, sucrose, sodium chloride and Tris) were purchased from VWR reagents and Sigma-Aldrich. L-α-phosphatidylcholine (EggPC, 840051P), cholesterol (700000P), 1,2-dioleoyl-sn-glycero-3-phosphoethanolamine (DOPE, 850725P), 1,2-dioleoyl-sn-glycero-3-phospho-L-serine (DOPS, 840035P) and L-α-phosphatidylinositol-4,5-bisphosphate [PI(4,5)P_2_, 840046P] were purchased from Avanti Polar Lipids. Bodipy TR ceramide (D-7540) was purchased from Invitrogen.

### Protein purification

Human septin octameric complexes containing SEPT2–GFP, SEPT6, SEPT7 and SEPT9i1 were co-expressed in *E. coli* and purified as described in detail elsewhere (Iv et al., 2021). Dark septin (without GFP tags) octamers were also produced following the same protocol. Briefly, septins were purified by nickel- histidine, streptavidin-biotin affinities and ion exchange chromatography. The septin complex mostly consisted of mixtures of the octamers SEPT2–GFP-SEPT6-SEPT7-SEPT9-SEPT9-SEPT7-SEPT6-SEPT2–GFP. Those complexes were conserved in highly salted solutions (300 mM KCl, 50 mM Tris, pH 8) at −80°C (after quick freeze in liquid nitrogen) to avoid polymerization and aggregation of complexes in solution. An SDS-PAGE gel showing the state of the protein complex is shown in Fig. S7A.

### GUV assay

GUVs were prepared by either platinum wire method (Beber et al., 2019a) or PVA-gel assisted methods (Beber et al., 2019a) with lipid mixtures of 56.5% EggPC, 15% cholesterol, 10% DOPE, 10% DOPS, 8% PI(4,5)P_2_ and 0.5% Bodipy TR ceramide. In both methods, lipids were resuspended in 10 mM Tris pH 7.8, 50 mM NaCl and the desired concentration of sucrose. Formed GUVs were collected and transferred in solution with 10 mM Tris pH 7.8 containing the desired concentration of NaCl. The osmolarity inside and outside of vesicles was adjusted using sucrose based on the NaCl concentration of external solution. Septins were added to GUVs from outside, and samples were incubated for more than 1 h. Samples were then transferred to the glass chambers passivated with 5% β-casein (wt), 10 mM Tris pH 7.8 and 75 mM NaCl, and were observed using confocal fluorescence microscopy. Confocal microscopy experiments were performed on a Nikon Eclipse TE2000 inverted microscope equipped with the software EZ-C1, and images were analyzed by ImageJ software. To obtain 3D images, an inverted Eclipse Ti-E (Nikon) confocal microscope equipped with spinning disk CSU-X1 (Yokogawa) and Live-SR (Gataca Systems) integrated with Metamorph software was used. Images were taken by Prime 95B (Photometrics) and were further analyzed using ImageJ.

### Cryo-EM and cryo-electron tomography

Lipid mixture [57% EggPC, 15% cholesterol, 10% DOPE, 10% DOPS and 8% PI(4,5)P_2_] dissolved in chloroform was quickly dried under nitrogen. Obtained lipid dried films were further dried under vacuum for more than 30 min. Lipids were resuspended in 10 mM Tris pH 7.8 buffer containing 75 mM NaCl by vortexing to obtain various size of LUVs. The LUV suspension was diluted and septins were added at a final concentration of 17 nM septin and 0.0125–0.025 g/l lipid. The mixture was incubated for 1 h, and 10 nm gold beads (Aurion, Electron Microscopy Science) were added to the solution. Then, 4 µl of the solution was deposited on a glow-discharged lacey carbon electron microscopy grid (Ted Pella, USA). Most of the solution was blotted away from the grid to leave a thin (<100 nm) film of aqueous solution. The blotting was carried out on the opposite side from the liquid drop and plunge frozen in liquid ethane at −181°C using an automated freeze plunging apparatus (EM GP, Leica, Germany). The samples were kept in liquid nitrogen and imaged using a Tecnai G2 (FEI, Eindhoven, Netherlands) transmission electron microscope operated at 200 kV and equipped with a 4000×4000 CMOS camera (F416, TVIPS). For cryo-electron tomography, tilt series were collected in low-dose mode every two degrees from 0 to −34°, then from +2 to 60°, and finally from −36 to −60° to minimize irradiation at the lowest angles. The dose per image was set at 1 electron per Å^2^. The imaging was performed at a magnification of 50,000. The consecutive images were aligned using the IMOD software (https://bio3d.colorado.edu/imod/) with the help of gold beads. Simultaneous iterative reconstruction technique (SIRT) reconstruction was carried out using for volumetric reconstitution. The segmentation was performed manually using IMOD.

### SEM

Wrinkled polydimethylsiloxane (PDMS) substrates having 1.6±0.1 µm period and 0.20±0.05 µm amplitude were designed and fabricated to achieve a pattern curvature of ±3 µm^−1^, or as described in a previous report for fabrication of a 1D wave (Pellegrino et al., 2020). Similarly, curvatures of 1.7 and 4.5 µm^−1^ were generated as well as flat patterns fabricated from PDMS baked on silicon wafers. Fabricated wavy PDMS substrates were used to make a surface replica using UV-curable adhesive (Norland Optical Adhesive NOA71 or NOA81). Small drops (∼ 5 µl) of NOA71 were placed on circular coverslips (12 mm diameter) and the wavy surface of the PDMS chip had contact with NOA71. NOA71 was cured under a UV lamp for 7 min and the PDMS chip was carefully pealed and stored for further replication. An SUV solution to prepare the supported lipid bilayer on top of the wavy substrate was prepared as follows: a desired amount of the lipid mixture [57% EggPC, 15% cholesterol, 10% DOPE, 10% DOPS and 8% PI(4,5)P_2_] dissolved in chloroform was quickly dried under nitrogen and the obtained lipid dried films were further dried under vacuum for more than 30 min; lipids were resuspended at 5 g/l in 20 mM citrate buffer at pH 4.8 containing 150 mM NaCl, and the suspension was sonicated for around 30 min until the solution became transparent; the obtained SUV suspension was kept in a −20°C freezer, and before use, it was diluted to 1 g/l with the same citrate buffer.

Next, 100 µL of 1 g/l SUV suspension was deposited on the prepared NOA71 wavy substrates treated with plasma (tabletop plasma cleaner) for 1–3 min in advance to ionize the surface. Samples were incubated for 1 h and the substrate was washed thoroughly four times using 20 mM citrate buffer at pH 4.8 containing 150 mM NaCl, and then four times using 10 mM Tris at pH 7.8 buffer containing 75 mM NaCl. After the washes, septins were added on the substrate at various concentrations in 10 mM Tris buffer at pH 7.8 containing 75 mM NaCl. After 1 h of incubation, samples were fixed with 2% glutaraldehyde in 0.1 M sodium cacodylate for 15 min. The samples were washed three times with 0.1 M sodium cacodylate pH 7.4 and incubated for 10 min with a second fixative (1% osmium tetroxide in 0.1 M sodium cacodylate). After three washes with water, the samples were incubated with 1% tannic acid for 10 min and subsequently washed three times with water before being incubated with uranyl acetate (1% in water) for 10 min. The samples were then dehydrated using baths with increasing ethanol concentrations (50, 70, 95 and 100%) and processed using a critical point dryer (CPD 300, Leica). After being mounted on a sample holder, the samples were coated with around 1 nm of either tungsten or platinum (ACE 200, Leica). SEM imaging was performed using a GeminiSEM 500 microscope (Zeiss, Germany). SEM images were segmented using Weka trainable segmentation on ImageJ and were further analyzed using OrientationJ to determine the orientation of the filaments. To extract the periodicities existing in the mesh-like organization of septin filaments, the probability map obtained as a result of segmentation was fast Fourier transformed (FFT) on ImageJ, and the square root of the raw power spectrum obtained by FFT was integrated by angles and displayed as a 1D power spectrum. The spectrum was fitted by a Lorentzian function to obtain the distance and full width at half maximum (FWHM).

### Fluorescence microscopy image analysis

#### Measurement of periodicity in GUV deformation

The fluorescence signals from the membranes of a deformed GUV (regular deformation or partially distorted deformation) were used for the measurement of periodicity in membrane deformation by septins. The image was binarized by thresholding and particle analysis in ImageJ. The GUV was fitted with an ellipsoid to determine the center of vesicles. The *x* and *y* coordinates of the vesicle perimeter were then obtained by using the Find Edge function in ImageJ. Distances from the center to a point (pixel) on the vesicle perimeter was obtained for each point of the vesicle perimeter and the distances were plotted as a function of the angle *θ* (Fig. S5). The angles *θ* were converted to the actual distances *d* by: *d*=2π*R*[(*θ*+180/360)×pixel size of the image (in μm/pixels)]. Here, the mean radius *R* was obtained according to the major and minor length of the fitted ellipsoid by (major length+minor length)/4. Local maxima were found under the condition where d>0.25 μm and minimum peak prominance >0.035 μm. Several images were subjected to this image analysis and all the distances *d* were plotted in a histogram.

### SEM image analysis

#### Displaying the orientation profiles on septin filaments

Raw SEM images were segmented by using machine-learning-based Weka trainable segmentation in ImageJ. The whole image was then classified into three categories [septin filaments, small vesicles bound on membranes and background (supported lipid bilayer only)] (an example is shown in Fig. S7B). A probability map was generated by segmentation (Fig. S7B). The resulting probability map for septin filaments was used for further image analysis. The probability map of septin filaments was first rotated so that the 1D periodic waves of substrates oriented perpendicularly. The ImageJ plugin OrientationJ displayed the orientation of the filaments on probability maps by color, and a spectrum displayed the angle distribution.

#### Measurement of the periodicity in septin mesh organization using 2D FFT signals

Raw SEM images were segmented and the probability maps of septin filaments were obtained as described above. From the whole probability map, sections where meshes were clearly visualized on convex regions (positive curvature) were cropped and then Fourier transformed by the 2D FFT function using ImageJ. The resulting 2D FFT signal was radially integrated using the ImageJ plugin ‘Radial_Profile_Angle’. The 1D spectra of the signals were then plotted in reciprocal nanometer (nm^−1^) (Fig. S3C). Several images were subjected to this analysis, and all the obtained 1D spectra were averaged by different positions assuming that the density of septin meshes are uniform over the sample.

### Model for Monte Carlo simulations

The local orientation of the nematic field, which models filament orientation, is denoted by the unit vector 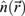, which lies in the local tangent plane of the membrane and is free to rotate in this plane. The vesicle is modeled by a closed, triangulated network of vertices (i=1, 2,… N) (Fig. S7C), and filament–membrane interactions are modelled as anisotropic spontaneous curvatures of the membrane in the vicinity of the filament. Two nematic fields, 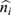 and 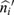, corresponding to the nematic field of the first and second layers, respectively, are at the same vertex ‘i’. Two fields prefer to orient perpendicular to each other via an interaction term 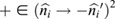, which preserves the 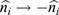 symmetry of the nematics. Nematics at the same layer tend to align with each other via the nearest-neighbor nematic interaction energy, commonly modeled by the Lebwohl–Lasher form 
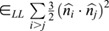
 (Lebwohl and Lasher, 1972). Here, ∈ _*LL*_ is the strength of the nematic interaction in one constant approximation (i.e. when splay and bend moduli are assumed to have the same value). The total energy functional is given by:

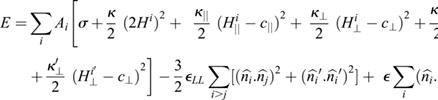


All the terms in the first set of square brackets are summed over surface elements *A*_*i*_ associated with the i-th vertex. The first term represents surface tension, and the second is the Helfrich elastic energy (Helfrich, 1973) for membranes with isotropic bending rigidity *κ* and membrane mean curvature *H*=(*c*_1_+*c*_2_)/2. Here, *c*_1_ and *c*_2_ are the local principal curvatures on the membrane surface along tangent vectors 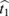 and 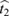, respectively. 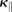 and *κ*_⊥_ are the induced membrane bending rigidities, 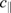 and *c*_⊥_ are the induced intrinsic curvatures along and perpendicular to 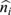 (or 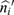 in the second layer), which are the orientations of the filaments in the local tangent planes in the two layers, respectively. Here, for human septins exhibiting intrinsic negative curvature, we set 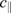 to a non-zero negative value and *c*_⊥_=0. The local membrane curvature along 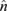 (and 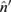, in the second layer) is given by 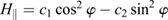 (and 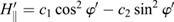) and that perpendicular to 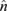 (and 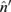) is given by *H*_⊥_ = *c*_1_ cos^2^ *ϕ*−*c*_2_ sin^2^ *ϕ* (and 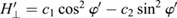), where *ϕ* and *ϕ*′ denote the angles between the principal direction 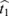 and the filament orientations 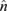 and 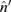, respectively. Note that at a given point on the membrane, the principal curvatures *c*_1_ and *c*_2_ and the corresponding eigen vectors 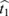 and 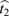, are unique, but the curvatures 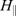 and 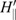, along the filaments 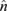 and 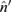, respectively, are different.

In a model for a fixed surface, the local mean curvature *H*^*i*^ at any point on it is predetermined, but the filaments seek out appropriate 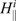 (and 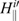 for the second layer) at any point by changing their orientation, such that they minimize the global energy of the system. In a model for a deformable GUV, however, the vertices are continuously moved during Monte Carlo steps, in order to explore the shape space of the deformable fluid surface. In our simulation, the septin concentration is limited by the number of vertices in the network (maximum of one in each layer). Also, our nematic interaction acts only between neighboring vertices and the energy is lowered if the neighbors are parallel to each other. Therefore, there is a tendency in the nematics to self-assemble even if the vertices are sparsely populated at the starting of the simulation. However, in reality, increased septin concentration in the solution decrease the inter-septin distance on the membrane. We modeled the increased concentration by increasing the strength of the septin–membrane interaction (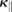).

### Approximate energy comparison between a smooth surface and valley or hill periodic deformation on fluid membranes at low septin concentrations in simulation

Here, we explain the results found in our simulation, in which a smooth spherical surface was obtained when the first and second layers interacted with the membrane in equal strength, i.e. 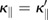, whereas valley/hill deformations were favorable when the second layer weakly interacted, i.e. 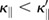 and 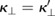. For a sphere (vesicle with radius *R*) of area *A*, the constant local curvature 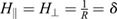, is a small number relative to 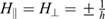 for a hill/valley. For a purely convex hill (for example, a hemisphere of radius *h*, with *h*<<*R*), the isotropic curvature 1/*h*>0, and, for a concave valley, −1/*h*<0. We assumed that the nematics in the two layers are perpendicular to each other (true at sufficiently high ε) and let the intrinsic curvature for septin be 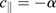, which is of the same order as *h*^−1^. Whereas for a sphere of area *A*, the Helfrich energy is 8π*κ*, for the undulated vesicle surface of approximately the same total area *A*, equally distributed between hills and valleys (i.e. ∼*A*/2 each), the Helfrich energy is higher, namely, 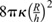. Furthermore, the energy cost from the anisotropic curvature terms, for a sphere, is: 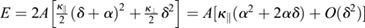. Compared to this, the undulated vesicle surface costs: 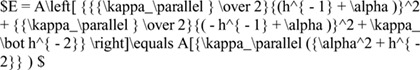 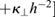, which is higher than that of the sphere, as *h*^−1^≫*δ*. The overall pre-factor 2 in both the expressions is because the second layer, where filament orientations are orthogonal to the first layer, gives the same contribution as the first layer. Note that on a hemisphere, 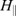 and *H*_⊥_ do not change with filament orientation. Furthermore, the nematic energy (*ε*_*LL*_) contribution of the undulated vesicle will be higher than that of the sphere, as surface undulation causes additional topological defects compared to that on a sphere. Note that, as the areas of the spherical vesicle and the deformed vesicle are close, their surface tension energies are not significantly different. Besides, the septin-coated membrane is expected to be stiffer than the bare bilayer membrane (for which *κ*∼10−20*k*_*b*_*T*, where *k_b_* is the Boltzmann constant (1.38×10^–23^ m^2^ kg s^–2^ K^–1^) and *T* is the temperature in Kelvin), we set 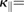
25*k*_*b*_*T*. We found that setting *κ*=0 or *κ*=10*k*_*b*_*T* in the simulation only made minor qualitative differences in the shape, in particular, valleys and hills became little flatter with non-zero *κ* as it opposed higher curvature.

## Supplementary Material

10.1242/joces.260813_sup1 Supplementary information

## References

[JCS260813C1] Addi, C., Bai, J. and Echard, A. (2018). Actin, microtubule, septin and ESCRT filament remodeling during late steps of cytokinesis. *Curr. Opin. Cell Biol.* 50, 27-34. 10.1016/j.ceb.2018.01.007 29438904

[JCS260813C2] Beber, A., Alqabandi, M., Prévost, C., Viars, F., Bassereau, P., Bertin, A., Mangenot, S., Beber, A., Alqabandi, M., Prévost, C. et al. (2019a). Septin-based readout of PI (4, 5) P2 incorporation into membranes of giant unilamellar vesicles. *Cytoskeleton (Hoboken)* 76, 92-103. 10.1002/cm.21480 30070077

[JCS260813C3] Beber, A., Taveneau, C., Nania, M., Tsai, F.-C., Di Cicco, A., Bassereau, P., Lévy, D., Cabral, J. T., Isambert, H., Mangenot, S. et al. (2019b). Membrane reshaping by micrometric curvature sensitive septin filaments. *Nat. Commun.* 10, 420. 10.1038/s41467-019-08344-5 30679428 PMC6345803

[JCS260813C4] Behera, A., Kumar, G., Akram, S. A. and Sain, A. (2021). Deformation of membrane vesicles due to chiral surface proteins. *Soft Mat.* 17, 7953-7962. 10.1039/D1SM00628B 34378621

[JCS260813C5] Bertin, A., McMurray, M. A., Grob, P., Park, S.-S., Garcia, G., Patanwala, I., Ng, H., Alber, T., Thorner, J. and Nogales, E. (2008). Saccharomyces cerevisiae septins: Supramolecular organization of heterooligomers and the mechanism of filament assembly. *Proc. Natl. Acad. Sci. USA* 105, 8274-8279. 10.1073/pnas.0803330105 18550837 PMC2426963

[JCS260813C6] Bertin, A., McMurray, M. A., Thai, L., Garcia, G., III, Votin, V., Grob, P., Allyn, T., Thorner, J. and Nogales, E. (2010). Phosphatidylinositol-4,5-bisphosphate promotes budding yeast septin filament assembly and organization. *J. Mol. Biol.* 404, 711-731. 10.1016/j.jmb.2010.10.002 20951708 PMC3005623

[JCS260813C7] Bertin, A., McMurray, M. A., Pierson, J., Thai, L., McDonald, K. L., Zehr, E. A., García, G., III, Peters, P., Thorner, J. and Nogales, E. (2012). Three-dimensional ultrastructure of the septin filament network in Saccharomyces cerevisiae. *Mol. Biol. Cell* 23, 423-432. 10.1091/mbc.e11-10-0850 22160597 PMC3268722

[JCS260813C8] Camarda, R., Williams, J. and Goga, A. (2017). In vivo reprogramming of cancer metabolism by MYC. *Front. Cell Dev. Biol* 5, 1-7. 10.3389/fcell.2017.00035 28443280 PMC5386977

[JCS260813C9] Cannon, K. S., Woods, B. L. and Gladfelter, A. S. (2017). The unsolved problem of how cells sense micron-scale curvature. *Trends Biochem. Sci.* 42, 961-976. 10.1016/j.tibs.2017.10.001 29089160 PMC5705049

[JCS260813C10] Cannon, K. S., Woods, B. L., Crutchley, J. M. and Gladfelter, A. S. (2019). An amphipathic helix enables septins to sense micrometer-scale membrane curvature. *J. Cell Biol.* 218, 1128-1137. 10.1083/jcb.201807211 30659102 PMC6446858

[JCS260813C11] Cao, L., Ding, X., Yu, W., Yang, X., Shen, S. and Yu, L. (2007). Phylogenetic and evolutionary analysis of the septin protein family in metazoan. *FEBS Lett.* 581, 5526-5532. 10.1016/j.febslet.2007.10.032 17967425

[JCS260813C12] Cao, L., Yu, W., Wu, Y. and Yu, L. (2009). The evolution, complex structures and function of septin proteins. *Cell. Mol. Life Sci. CMLS* 66, 3309-3323. 10.1007/s00018-009-0087-2 19597764 PMC11115805

[JCS260813C13] Castro, D. K. S. d. V., da Silva, S. M. d. O., Pereira, H. D., Macedo, J. N. A., Leonardo, D. A., Valadares, N. F., Kumagai, P. S., Brandão-Neto, J., Araújo, A. P. U. and Garratt, R. C. (2020). A complete compendium of crystal structures for the human SEPT3 subgroup reveals functional plasticity at a specific septin interface. *IUCrJ* 7, 462-479. 10.1107/S2052252520002973 PMC7201284 32431830

[JCS260813C14] Caudron, F. and Barral, Y. (2009). Septins and the lateral compartmentalization of eukaryotic membranes. *Dev. Cell* 16, 493-506. 10.1016/j.devcel.2009.04.003 19386259

[JCS260813C15] de Almeida Marques, I., Valadares, N. F., Garcia, W., Damalio, J. C. P., Macedo, J. N. A., de Araújo, A. P. U., Botello, C. A., Andreu, J. M. and Garratt, R. C. (2012). Septin C-terminal domain interactions: implications for filament stability and assembly. *Cell Biochem. Biophys* 62, 317-328. 10.1007/s12013-011-9307-0 22001952

[JCS260813C16] Ewers, H., Tada, T., Petersen, J. D., Racz, B., Sheng, M. and Choquet, D. (2014). A septin-dependent diffusion barrier at dendritic spine necks. *PLoS ONE* 9, e113916. 10.1371/journal.pone.0113916 25494357 PMC4262254

[JCS260813C17] Franquelim, H. G., Dietz, H. and Schwille, P. (2021). Reversible membrane deformations by straight DNA origami filaments. *Soft Mat.* 17, 276-287. 10.1039/D0SM00150C 32406895

[JCS260813C18] Garcia, G., III, Bertin, A., Li, Z., Song, Y., McMurray, M. A., Thorner, J. and Nogales, E. (2011). Subunit-dependent modulation of septin assembly: budding yeast septin Shs1 promotes ring and gauze formation. *J. Cell Biol.* 195, 993-1004. 10.1083/jcb.201107123 22144691 PMC3241732

[JCS260813C19] Gilden, J. K., Peck, S., Chen, Y.-C. M. and Krummel, M. F. (2012). The septin cytoskeleton facilitates membrane retraction during motility and blebbing. *J. Cell Biol.* 196, 103-114. 10.1083/jcb.201105127 22232702 PMC3255977

[JCS260813C20] Hall, P. A. and Russell, S. E. H. (2004). The pathobiology of the septin gene family. *J. Pathol* 204, 489-505. 10.1002/path.1654 15495264

[JCS260813C21] Helfrich, W. (1973). Elastic properties of lipid bilayers: theory and possible experiments. *Z. Naturforschung Teil C Biochem. Biophys. Biol. Virol* 28, 693-703. 10.1515/znc-1973-11-1209 4273690

[JCS260813C22] Hu, Q., Milenkovic, L., Jin, H., Scott, M. P., Nachury, M. V., Spiliotis, E. T. and Nelson, W. J. (2010). A septin diffusion barrier at the base of the primary cilium maintains ciliary membrane protein distribution. *Science* 329, 436-439. 10.1126/science.1191054 20558667 PMC3092790

[JCS260813C23] Ihara, M., Kinoshita, A., Yamada, S., Tanaka, H., Tanigaki, A., Kitano, A., Goto, M., Okubo, K., Nishiyama, H., Ogawa, O. et al. (2005). Cortical organization by the septin cytoskeleton is essential for structural and mechanical integrity of mammalian spermatozoa. *Dev. Cell* 8, 343-352. 10.1016/j.devcel.2004.12.005 15737930

[JCS260813C25] Iv, F., Martins, C. S., Castro-Linares, G., Taveneau, C., Barbier, P., Verdier-Pinard, P., Camoin, L., Audebert, S., Tsai, F.-C., Ramond, L. et al. (2021). Insights into animal septins using recombinant human septin octamers with distinct SEPT9 isoforms. *J. Cell Sci.* 134, jcs258484. 10.1242/jcs.258484 34350965

[JCS260813C26] Karasmanis, E. P., Hwang, D., Nakos, K., Bowen, J. R., Angelis, D. and Spiliotis, E. T. (2019). A septin double ring controls the spatiotemporal organization of the ESCRT machinery in cytokinetic abscission. *Curr. Biol.* 29, 2174-2182.e7. 10.1016/j.cub.2019.05.050 31204162 PMC6620605

[JCS260813C27] Kinoshita, M. (2003). Assembly of mammalian septins. *J. Biochem. (Tokyo)* 134, 491-496. 10.1093/jb/mvg182 14607974

[JCS260813C28] Kumar, G., Ramakrishnan, N. and Sain, A. (2019). Tubulation pattern of membrane vesicles coated with biofilaments. *Phys. Rev. E* 99, 022414. 10.1103/PhysRevE.99.022414 30934309

[JCS260813C29] Kumar, G., Duggisetty, S. C. and Srivastava, A. (2022). A review of mechanics-based mesoscopic membrane remodeling methods: capturing both the physics and the chemical diversity. *J. Membr. Biol.* 255, 757-777. 10.1007/s00232-022-00268-4 36197492

[JCS260813C30] Lebwohl, P. A. and Lasher, G. (1972). Nematic-liquid-crystal order—a monte carlo calculation. *Phys Rev A* 6, 426-429. 10.1103/PhysRevA.6.426

[JCS260813C31] Leonardo, D. A., Cavini, I. A., Sala, F. A., Mendonça, D. C., Rosa, H. V. D., Kumagai, P. S., Crusca, E. J., Valadares, N. F., Marques, I. A., Brandão-Neto, J. et al. (2021). Orientational ambiguity in septin coiled coils and its structural basis. *J. Mol. Biol.* 433, 166889. 10.1016/j.jmb.2021.166889 33639214

[JCS260813C32] Lindsey, R. and Momany, M. (2006). Septin localization across kingdoms: three themes with variations. *Curr. Opin. Microbiol* 9, 559-565. 10.1016/j.mib.2006.10.009 17067846

[JCS260813C33] Martins, C. S., Taveneau, C., Castro-Linares, G., Baibakov, M., Buzhinsky, N., Eroles, M., Milanović, V., Omi, S., Pedelacq, J.-D., Iv, F. et al. (2023). Human septins organize as octamer-based filaments and mediate actin-membrane anchoring in cells. *J. Cell Biol.* 222, e202203016. 10.1083/jcb.202203016 36562751 PMC9802686

[JCS260813C34] Mavrakis, M., Azou-Gros, Y., Tsai, F.-C., Alvarado, J., Bertin, A., Iv, F., Kress, A., Brasselet, S., Koenderink, G. H. and Lecuit, T. (2014). Septins promote F-actin ring formation by crosslinking actin filaments into curved bundles. *Nat. Cell Biol.* 16, 322-334. 10.1038/ncb2921 24633326

[JCS260813C35] Mendonça, D. C., Guimarães, S. L., Pereira, H. D., Pinto, A. A., de Farias, M. A., de Godoy, A. S., Araujo, A. P. U., van Heel, M., Portugal, R. V. and Garratt, R. C. (2021). An atomic model for the human septin hexamer by cryo-EM. *J. Mol. Biol.* 433, 167096. 10.1016/j.jmb.2021.167096 34116125

[JCS260813C36] Mim, C. and Unger, V. M. (2012). Membrane curvature and its generation by BAR proteins. *Trends Biochem. Sci.* 37, 526-533. 10.1016/j.tibs.2012.09.001 23058040 PMC3508348

[JCS260813C37] Mostowy, S. and Cossart, P. (2012). Septins: The fourth component of the cytoskeleton. *Nat. Rev. Mol. Cell Biol.* 13, 183-194. 10.1038/nrm3284 22314400

[JCS260813C38] Nania, M., Matar, O. K. and Cabral, J. T. (2015). Frontal vitrification of PDMS using air plasma and consequences for surface wrinkling. *Soft Mat.* 11, 3067-3075. 10.1039/C4SM02840F 25742777

[JCS260813C39] Nania, M., Foglia, F., Matar, O. K. and Cabral, J. T. (2017). Sub-100 nm wrinkling of polydimethylsiloxane by double frontal oxidation. *Nanoscale* 9, 2030-2037. 10.1039/C6NR08255F 28106209

[JCS260813C40] Osawa, M., Anderson, D. E. and Erickson, H. P. (2009). Curved FtsZ protofilaments generate bending forces on liposome membranes. *EMBO J.* 28, 3476-3484. 10.1038/emboj.2009.277 19779463 PMC2782090

[JCS260813C41] Patzig, J., Erwig, M. S., Tenzer, S., Kusch, K., Dibaj, P., Möbius, W., Goebbels, S., Schaeren-Wiemers, N., Nave, K.-A. and Werner, H. B. (2016). Septin/anillin filaments scaffold central nervous system myelin to accelerate nerve conduction. *eLife* 5, e17119. 10.7554/eLife.17119 27504968 PMC4978525

[JCS260813C42] Pellegrino, L., Khodaparast, S. and Cabral, J. T. (2020). Orthogonal wave superposition of wrinkled, plasma oxidised, polydimethylsiloxane surfaces. *Soft Mat.* 16, 595-603. 10.1039/C9SM02124H 31776531

[JCS260813C43] Ramakrishnan, N., Sunil Kumar, P. B. and Ipsen, J. H. (2013). Membrane-mediated aggregation of curvature-inducing nematogens and membrane tubulation. *Biophys. J.* 104, 1018-1028. 10.1016/j.bpj.2012.12.045 23473484 PMC3870796

[JCS260813C44] Shen, Y.-R., Wang, H.-Y., Kuo, Y.-C., Shih, S.-C., Hsu, C.-H., Chen, Y.-R., Wu, S.-R., Wang, C.-Y. and Kuo, P.-L. (2017). SEPT12 phosphorylation results in loss of the septin ring/sperm annulus, defective sperm motility and poor male fertility. *PLoS Genet.* 13, e1006631. 10.1371/journal.pgen.1006631 28346465 PMC5386304

[JCS260813C45] Shuman, B. and Momany, M. (2022). Septins from protists to people. *Front. Cell Dev. Biol.* 9, 824850. 10.3389/fcell.2021.824850 35111763 PMC8801916

[JCS260813C46] Spiliotis, E. T. (2018). Spatial effects-site-specific regulation of actin and microtubule organization by septin GTPases. *J. Cell Sci.* 131, jcs207555. 10.1242/jcs.207555 29326311 PMC5818061

[JCS260813C47] Surka, M. C., Tsang, C. W. and Trimble, W. S. (2002). The mammalian septin MSF localizes with microtubules and is required for completion of cytokinesis. *Mol. Biol. Cell* 13, 3532-3545. 10.1091/mbc.e02-01-0042 12388755 PMC129964

[JCS260813C48] Szuba, A., Bano, F., Castro Linares, G., Iv, F., Mavrakis, M., Richter, R. P., Bertin, A. and Koenderink, G. H. (2021). Membrane binding controls ordered self-assembly of animal septins. *eLife* 10, e63349. 10.7554/eLife.63349 33847563 PMC8099429

[JCS260813C49] Tada, T., Simonetta, A., Batterton, M., Kinoshita, M., Edbauer, D. and Sheng, M. (2007). Role of Septin cytoskeleton in spine morphogenesis and dendrite development in neurons. *Curr. Biol.* 17, 1752-1758. 10.1016/j.cub.2007.09.039 17935993 PMC2194646

[JCS260813C50] Tamborrini, D. and Piatti, S. (2019). Septin clearance from the division site triggers cytokinesis in budding yeast. *Microb. Cell* 6, 295-298. 10.15698/mic2019.06.681 31172014 PMC6545438

[JCS260813C51] Tanaka-Takiguchi, Y., Kinoshita, M. and Takiguchi, K. (2009). Septin-mediated uniform bracing of phospholipid membranes. *Curr. Biol.* 19, 140-145. 10.1016/j.cub.2008.12.030 19167227

[JCS260813C52] Zhang, J., Kong, C., Xie, H., McPherson, P. S., Grinstein, S. and Trimble, W. S. (1999). Phosphatidylinositol polyphosphate binding to the mammalian septin H5 is modulated by GTP. *Curr. Biol.* 9, 1458-1467. 10.1016/S0960-9822(00)80115-3 10607590

