# Supplementary material for: A human septin octamer complex sensitive to membrane curvature drives membrane deformation with a specific mesh-like organization
Source: J Cell Sci. 2023 Jun 12;136(11):jcs260813. doi: 10.1242/jcs.260813 (PMC10281262; doi:10.1242/jcs.260813)
Supplement: Supplementary information [file joces-136-260813-s1.pdf]

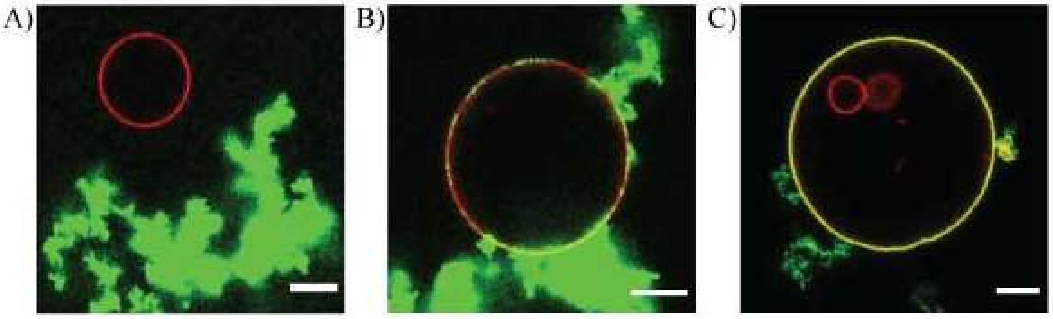

Fig. S1. Optimization of the septin/membrane interactions

(A) Typical confocal microscopy image showing the Interaction of GUV without any negatively charged lipids (lipid composition of GUV: EggPC 99.5%, Bodipy TR ceramide 0.5%) in 10 mM Tris pH 7.8 buffer containing 70mM of NaCl. Concentration of human septins octamers-GFP in solution outside GUVs is 260 nM.

(B) Typical confocal microscopy image of GUV containing DOPS and without PI(4,5)P_2 lipids (lipid composition of GUV: EggPC 64,5%, Cholesterol 15%, DOPS 10%, DOPE 10%, Bodipy TR ceramide 0.5%) in the presence of 70 mM NaCl, Tris 10 mM pH 7.8. Concentration of human septins octamers-GFP in solution outside GUV is 260 nM.

(C) Typical confocal microscopy image of GUV containing both DOPS and PI(4,5)P_2 lipids (lipid composition of GUV: EggPC 56,5%, Cholesterol 15%, DOPS 10%, DOPE 10%, PI(4,5)P_2 8%, Bodipy TR cermide 0.5%) in the presence of 70 mM NaCl, Tris 10 mM pH 7.8. Concentration of human septins octamers-GFP in solution outside GUV is 260 nM.

Scale bars: 5 μm

Journal of Cell Science • Supplementary information

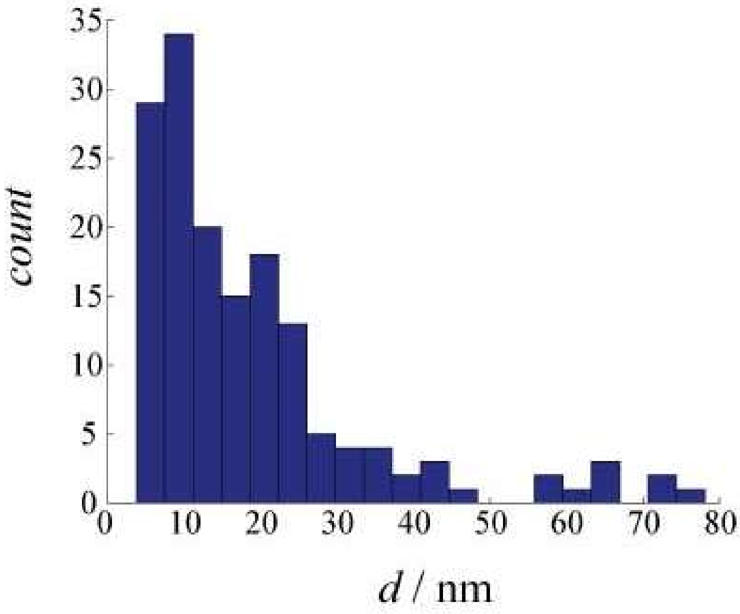

Fig. S2. Histogram of measured distances of adjacent septins filaments. *d* = 18±15 nm (Mean + SD) observed on 4 different tomograms of LUVs (157 counts, $N_{vesicle}$ = 4). Measurement was done with the segmented images.

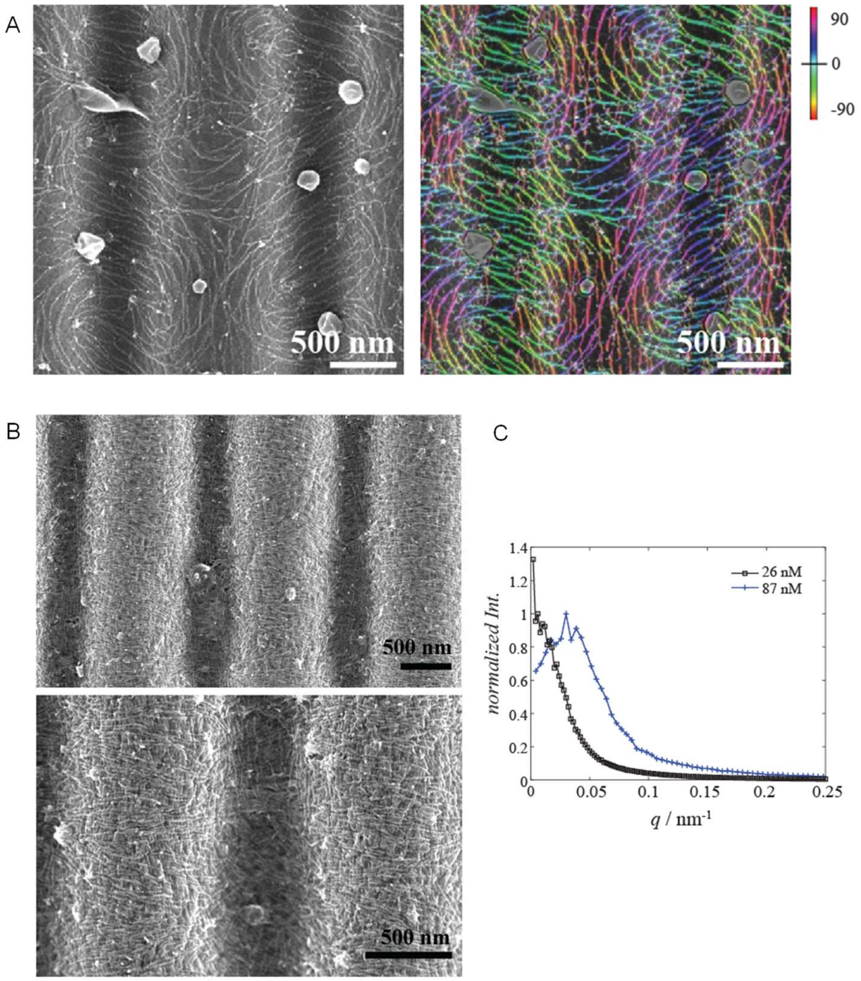

Fig. S3. SEM images of septins bound to lipid bilayers on wavy substrates. A Images for SEM observation of septins filaments on lipid bilayer supported by undulated solid substrates with 1.6 μm of periodicity and 0.20 μm of amplitude. Septins concentration is 26 nM in octamer. Raw image is shown on the left. On the right image, segmented septins filaments by using Trainable Weka Segmentation in ImageJ with color map showing the orientation of those filaments obtained by OrientationJ in ImageJ is superimposed to the Raw image. Spiral organization of septins filaments centered at concave regions are observed. **B** SEM image of septins filaments formed at 44 nM of septins in octamers on lipid bilayer supported by undulated solid substrates with 1.6 μm of periodicity and 0.20 μm of amplitude. Septin's mesh network was observed together with small vesicles bound to the surface. **C** Spectra obtained Radial integration of 2D FFT signals from septins mesh structures on convex regions in SEM images. Radial integration was done by using ImageJ plugin. "Radial_Profile_Angle". Integration was done in full angle

Journal of Cell Science • Supplementary information

(360 degrees), and spectra obtained from several images taken at different position in one sample (11 positions for 26 nM and 3 positions for 87 nM) were averaged assuming that the concentration of septins mesh are uniform over the sample. Spectra obtained at different concentration of septins in solution (black square: 26 nM, blue cross: 87 nM) are plotted as a function of q (nm^{-1}).

Journal of Cell Science • Supplementary information

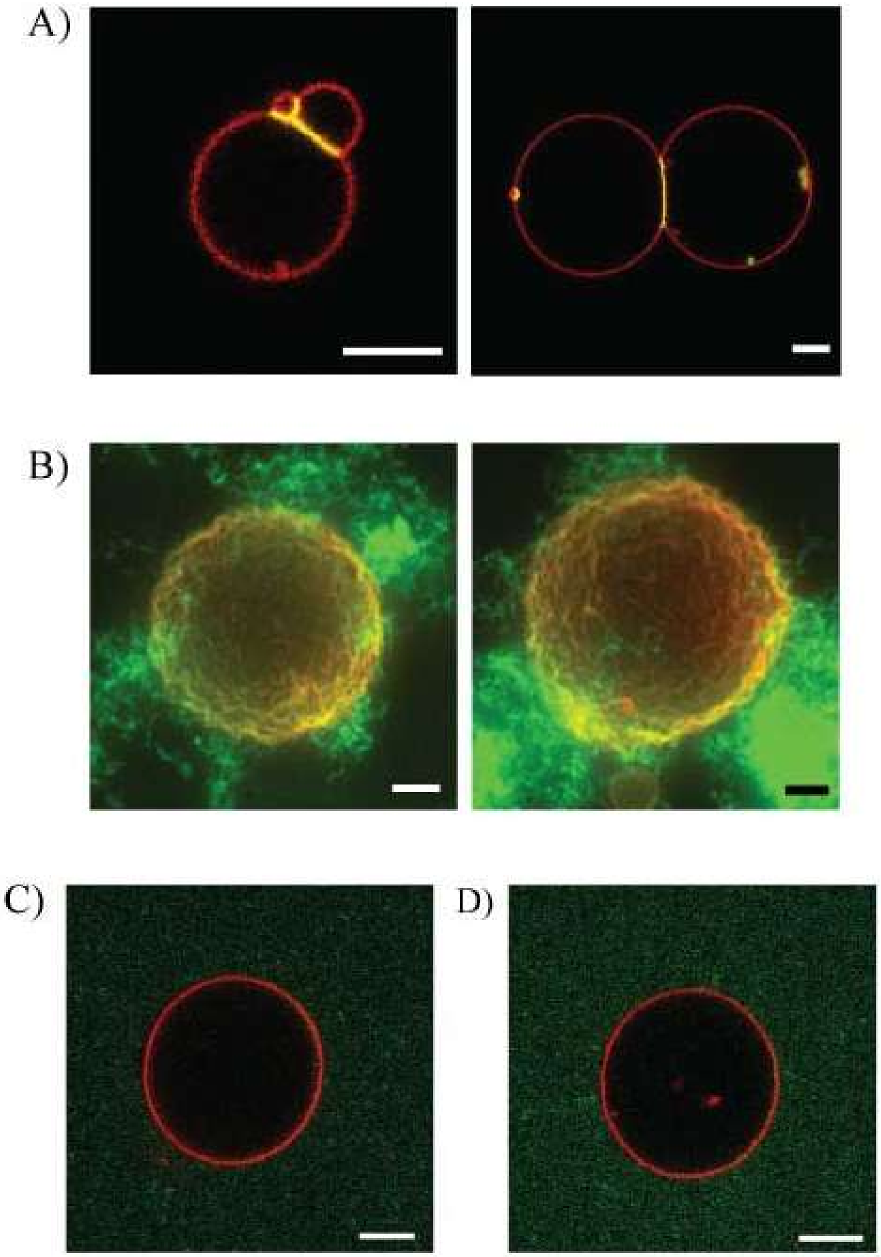

Fig. S4. Confocal images of GUVs interacting with varying septin concentrations. (A) Confocal microscopy images recorded at the equator of GUVs (lipid composition: EggPC 56.5%, Cholesterol 15%, DOPS 10%, DOPE 10%, PI(4,5)P2 8%, Bodipy TR cermide 0.5%) in the presence of 0.78 nM of human septins octamer-GFP in 70mM NaCl, 10 mM Tris pH 7.8.

(B) 3D reconstruction of confocal microscopy images of GUVs with 520 nM of human septins octamer-GFP in 70mM NaCl, 10 mM Tris pH 7.8.

Journal of Cell Science • Supplementary information

(C) A confocal microscopy image of a GUV (lipid composition: EggPC 99.5%, Bodipy TR ceramide 0.5%) with 260 nM of human septins octamer in 10 mM Tris pH 7.8 buffer containing 250 mM NaCl.
(D) A confocal microscopy image of a GUV (lipid composition: EggPC 64,5%, Cholesterol 15%, DOPS 10%, DOPE 10%, Bodipy TR cermide 0.5%) with 260 nM of human septins octamer in 10 mM Tris pH 7.8 buffer containing 250 mM NaCl. Scale bar: 5 µm.

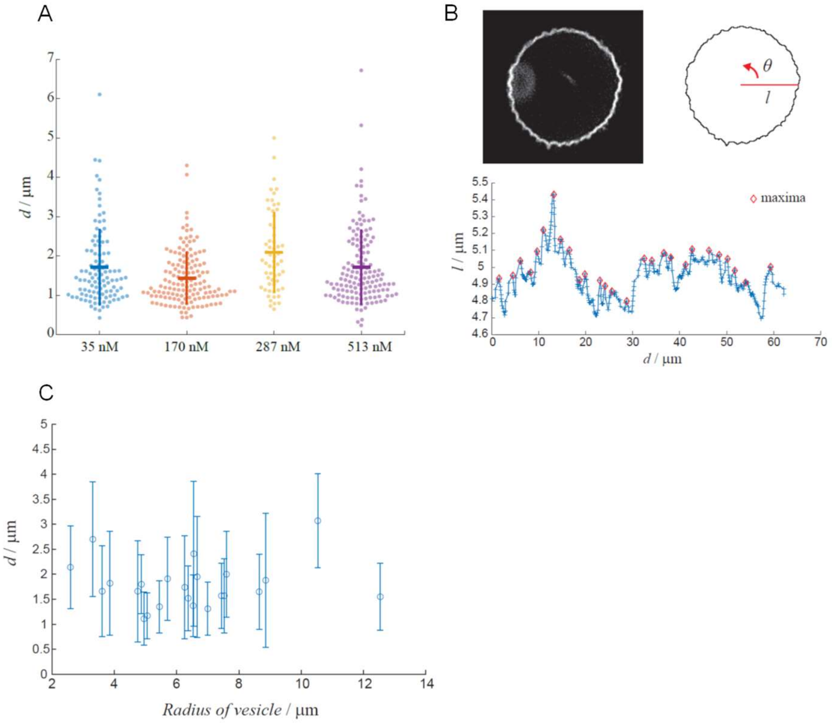

Fig. S5. Quantitative analysis of GUVS deformation features. A Dependency of distance between peaks of bumps in deformed GUVs on bulk concentration of human septins octamers (nM in octamer). 35 nM of dark human septins (1.7±1.0 µm (mean±SD), 106 counts, $N_{vesicle}$ = 5), 170 nM of human septins-GFP (1.4±0.7 µm (mean±SD), 143 counts, $N_{vesicle}$ = 5), 287 nM of human septins-GFP (2.1±1.9 µm (mean±SD), 92 counts, $N_{vesicle}$ = 3), 513 nM of human septins-GFP (1.9±1.3 µm (mean ±SD), 181 counts, $N_{vesicle}$ = 6). Perpendicular and horizontal bars show standard deviation (SD) and mean, respectively.

B Image of deformed vesicles (top left) and example of image analysis to obtain length *l* (µm) against the angle θ (top right) according to the x y coordinates of center and perimeter of a vesicle. The angle was converted to the actual distance d by $d = 2\pi R((\theta + 180)/360) \times pixel\ size\ (\mu m/pixel)$. Here, *R* is mean radius obtained according to the major and minor length of fitted ellipsoid by (major + minor)/4. Obtained *l* was plotted as a function of *d* (µm) shown in the bottom graph, where obtained local maxima were indicated as red diamonds. **C** Distance between each bumps as a function of the GUVs radius. Dependency of distance between peaks of bumps d in deformed GUVs on radius of vesicle. Distance d with standard deviation of deformed GUV in various bulk concentration of human septins octamers (nM in octamer) was plotted.

Journal of Cell Science • Supplementary information

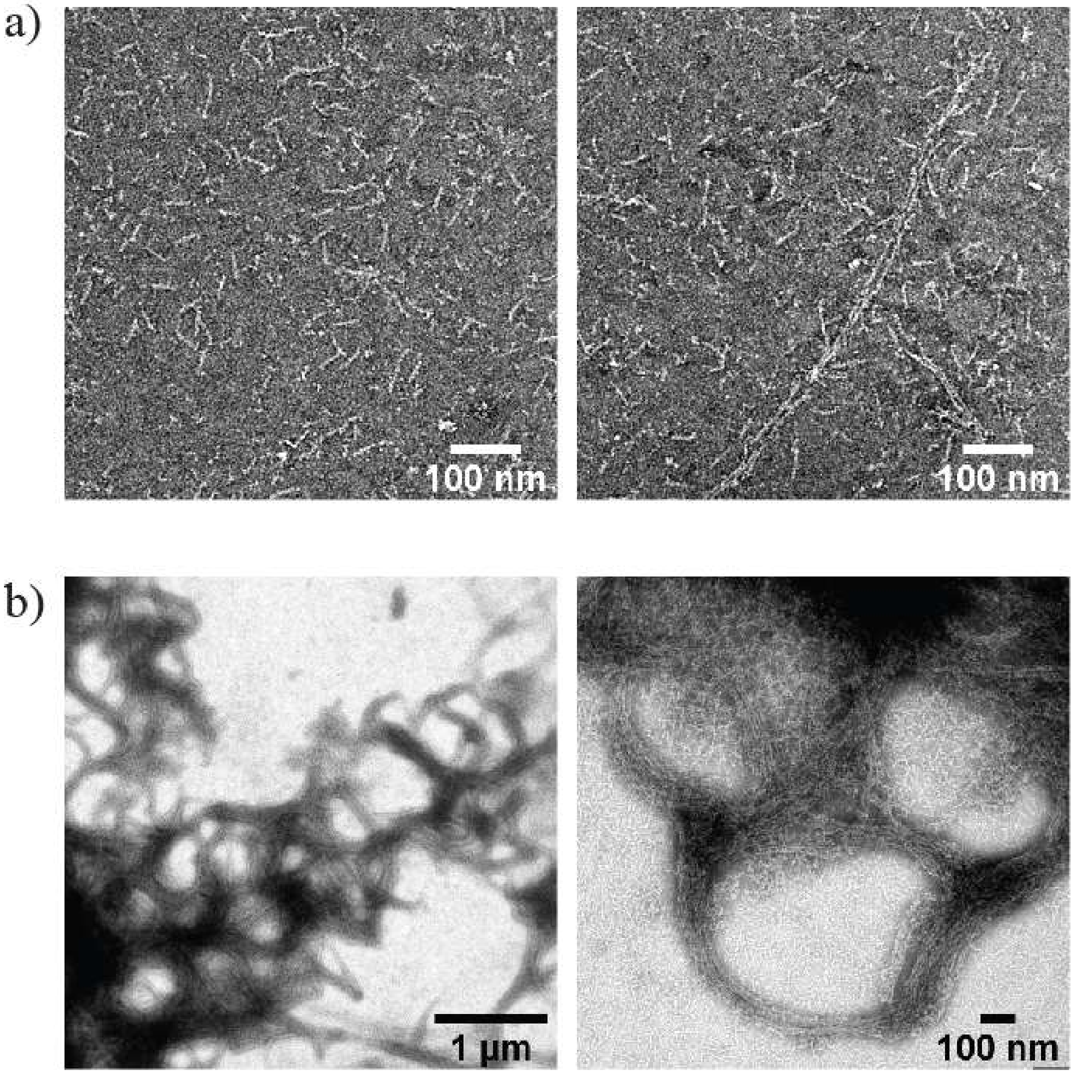

Fig. S6. Transmission electron microscopy images of human septins octamer-GFP in 50 mM Tris pH 8 containing 300 mM KCl (a) and human septins octamer-GFP after 1h incubation in 10 mM Tris pH 7.8 containing 75 mM NaCl (b). 4 µL of 0.02 mg/mL for high salt conditions (50 mM Tris pH 8 containing 300 mM KCl) (a) and 0.1 mg/mL for low salt condition (10 mM Tris pH 7.8 containing 75 mM NaCl) (b) were adsorbed for 30s on a carbon-coated electron microscopy grid (Electron Microscopy Sciences, CF300-CU). Grids were then rinsed and negatively stained for 1 min using 1% w/v uranyl formate, and liquids were blotted away with papers and girds were further dried in air. Images were collected using a Tecnai Spirit microscope (Thermo Scientific, FEI) operated at an acceleration voltage of 80kV and equipped with a Quemesa camera (Olympus).

Journal of Cell Science • Supplementary information

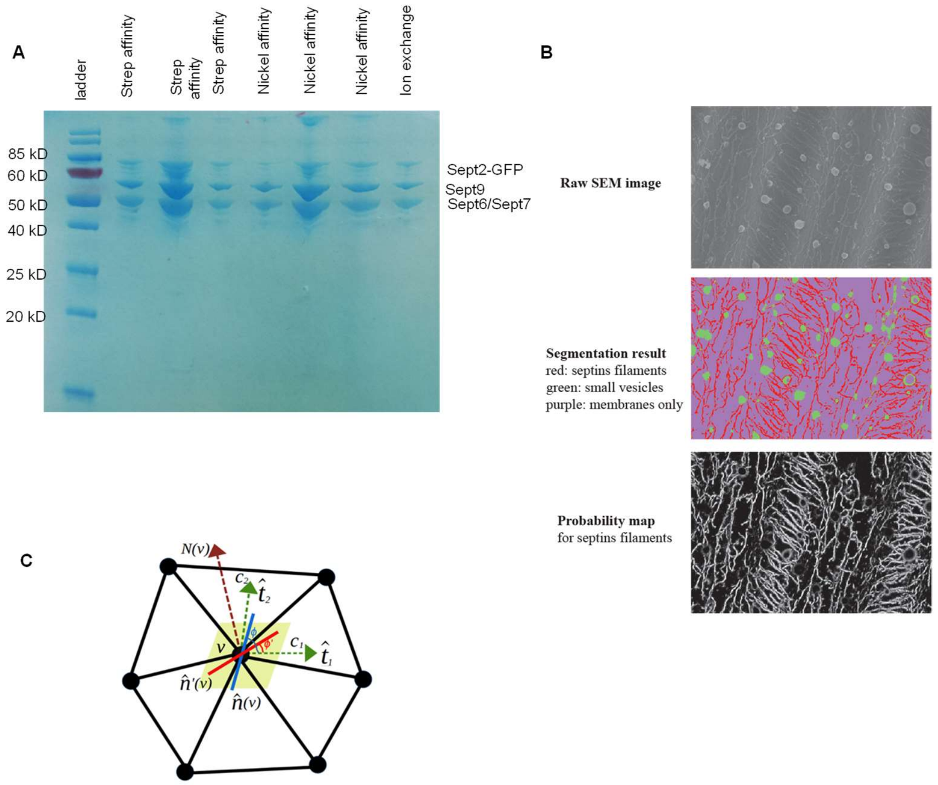

Fig. S7. Protein analysis, SEM segmentation procedure and description of the formalisation used to describe the septin nematic organization. A SDS-PAGE gel displaying the state of the septin complex in the course of the purification. **B** An example of segmentation of a SEM image. Raw image (top) and result of segmentation classified into three categories (septins filaments, small vesicles and membrane only) (middle) and probability map of septins filaments (bottom) by using Weka trainable segmentation in ImageJ software.

C Schematic representation of a small patch of membrane discretized by a network of vertices (solid black circles). At any vertex *v* the two layers of proteins are modelled by nematic directors $\hat{n}$ and $\hat{n}'$, shown by the blue and red colored rods, respectively, lying on the local tangent plane (in yellow) and characterized by the surface normal N(*v*). c_1 and c_2 are the local principal curvatures at *v* on the membrane surface along orthogonal tangent vectors $_1$ and $\hat{t}_2$,

while φ and φ' denote the angles between the principal direction $\hat{t}_1$ and the filament orientations $\hat{n}$ and $\hat{n}'$, respectively.

Journal of Cell Science • Supplementary information

Table S1. Surface coverage (%) of undulated supported lipid bilayers by human septins octamer at 8.7 nM and 26 nM of concentration in human septins octamer in solution. Values were obtained by analysis of SEM images where septins filaments were absorbed on lipid bilayer supported by undulated solid substrates with 1.6 μm of periodicity and 0.20 μm of amplitude. SEM images were segmented by using Weka trainable segmentation on imageJ, and probability map of septins filaments were further subjected by thresholding. Threshold image was used to measure the coverage of surface by septins. Measurement was done in 60% of the area near the center of the convex (Positive) or concave (Negative) part, corresponding to positive and negative curvatures, respectively. At 26 nM of septins, we have found roughly two ways of distribution: (1) the place where orientation of filaments is distinctively partitioned, and (2) the place where circular pattern and mesh structures by additional layer of septins were observed. The value corresponds to the mean ± standard deviation obtained by analysis of several regions (3 regions for 8.7 nM, 6 regions for 26 nM (1) and 13 regions for 26 nM (2)).

septins conc.	Negative	Positive
8.7 nM	32.2 ± 2.6	12.4 ± 2.1
26 nM	45.1 ± 4.8	28.8 ± 5.5
26 nM (2)	50.4 ± 4.6	53.0 ± 3.4

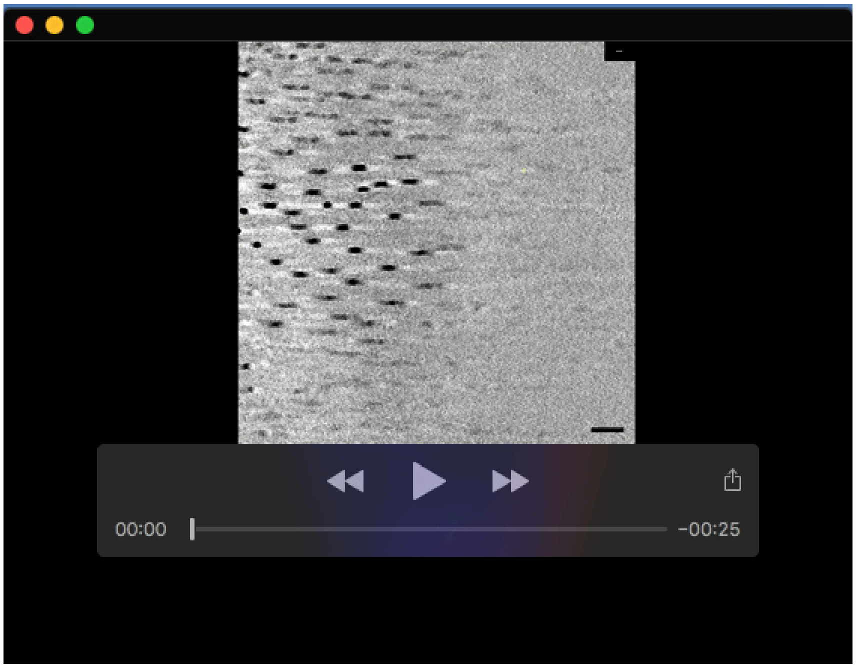

Movie 1. Slices within the cryo-tomogram from Figure 1.C displaying septin filaments bound to a liposome. The layers of filaments are visualized on different slices at t=2-4 seconds and t=29-31 seconds. Scale bar:100nm.
