# Supplementary material for: A human septin octamer complex sensitive to membrane curvature drives membrane deformation with a specific mesh-like organization
Source: J Cell Sci. 2023 Jun 12;136(11):jcs260813. doi: 10.1242/jcs.260813 (PMC10281262; doi:10.1242/jcs.260813)
Supplement: Supplementary file 1 [file joces-136-260813_review_history.pdf]

A human septin octamer complex sensitive to membrane curvature drives membrane deformation with a specific mesh-like organization

Koyomi Nakazawa, Gaurav Kumar, Brieuc Chauvin, Aurélie Di Cicco, Luca Pellegrino, Michael Trichet, Bassam Hajj, João Cabral, Anirban Sain, Stéphanie Mangenot and Aurelie Bertin
DOI: 10.1242/jcs.260813

Editor: Michael Way

Review timeline

Original submission:	16 November 2022
Editorial decision:	19 December 2022
First revision received:	11 April 2023
Editorial decision:	2 May 2023
Second revision received:	3 May 2023
Accepted:	4 May 2023

Original submission

First decision letter

MS ID#: JOCES/2022/260813

MS TITLE: A specific mesh-like organization of human septin octameric complex drives membrane reshaping and curvature sensitivity.

AUTHORS: Koyomi Nakazawa, Gaurav Kumar, Brieuc Chauvin, Aurélie Di Cicco, Luca Pellegrino, Michael Trichet, Bassam Hajj, João Cabral, Anirban Sain, Stéphanie Mangenot, and Aurelie Bertin
ARTICLE TYPE: Research Article

We have now reached a decision on the above manuscript.

To see the reviewers' reports and a copy of this decision letter, please go to: https://submit-jcs.biologists.org and click on the 'Manuscripts with Decisions' queue in the Author Area. (Corresponding author only has access to reviews.)

As you will see, the reviewers raise a number of substantial criticisms that prevent me from accepting the paper at this stage. They suggest, however, that a revised version might prove acceptable, if you can address their concerns. If you think that you can deal satisfactorily with the criticisms on revision, I would be pleased to see a revised manuscript. We would then return it to the reviewers.

Please ensure that you clearly highlight all changes made in the revised manuscript. Please avoid using 'Tracked changes' in Word files as these are lost in PDF conversion.

I should be grateful if you would also provide a point-by-point response detailing how you have dealt with the points raised by the reviewers in the 'Response to Reviewers' box. Please attend to all of the reviewers' comments. If you do not agree with any of their criticisms or suggestions please explain clearly why this is so.

© 2023. Published by The Company of Biologists under the terms of the Creative Commons Attribution License (https://creativecommons.org/licenses/by/4.0/).

Reviewer 1

Advance summary and potential significance to field

In this study, Nakazawa et al. describe the structural organization of human septins on membranes and examine their curvature sensitivity. With a wide set of techniques that span the study of this protein from the nanometer to the micron scale, the authors revealed the distribution of septin filaments into a 2-layer mesh of orthogonally ordered filaments and studied their curvature sensitivity and effect on free standing membranes. This is a very intriguing observation, as the studies on yeast have shown a contrasting assembly on these filaments. The model for network assembly they propose here is an interesting contribution to the field and opens many questions on the membrane reshaping capabilities of these proteins on reconstituted lipid membranes.

Comments for the author

The techniques and model together provide good understanding of how these filaments assemble and with which concentration thresholds deformations are enabled, this in a system that has not been sufficiently studied previously. The tomography also adds good evidence of superposed filaments and potential membrane deforming.

However, it seems that the curvature sensing is not defined very clearly and the range of the described specificity seems unclear. It may be useful to detect this behavior in different ranges of curvatures to build a stronger biophysical understanding of these interesting networks. SUV extrusion or tube pulling may be methods of choice to acquire this information.

It would be helpful to provide supplementary evidence that one layer interacts with both, membrane and the second layer, but that the second layer only interacts with the first layer. Additionally, a comment on how the second layer composed of the same septin subunits may achieve this selective binding seems to be appropriate.

There is no mention of the behavior of septins on flat surfaces such as supported lipid bilayers for example. It would be helpful to compare such experiments with the results in Figure 2.

Specific major points
- The Result section necessitates further editing, the authors should work on sentence structure and punctuation to ease reading.
- Figure 1 is missing panel labels that the result section mention.
- Are there any mesh configuration differences in SUVs that present deformations when compared to septin meshes on spherical SUVs? Are those deformations correlated to the size of the SUV? It would be interesting to yield a deeper analysis on the segmentation performed on those deformed SUVs as confinement and different vesicle curvature might induce changes in filament orientation or distribution.
- Can the authors provide information on the distribution of filaments on different surface curvatures? As human septins show a very different organization than the ones from budding yeast, further assessment of their intrinsic curvature should be performed with other patterned structures.
- Figure 4.B-6 shows a 3D reconstruction with visible septin signal in long structures surrounding the GUV. Is the protein inducing membrane tubulations in those areas? Or is it the result of the membrane being osmotically deflated and therefore showing unspecific protein binding? This should be checked by a negative control.
- The coarse grain models support the observations of the two-layers mesh organization and fit the experimental data but they do not provide valuable information to aid on the assembly of the model proposed.

Minor points
- The title may need tweaking (example: “A human curvature sensitive septin octamer complex drives membrane deformation with a specific mesh-like organization”).
- Introduction: line 3; actin and microtubules | line 6; cell motility and cell compartmentalization | line 8; Septin mis-regulation | line 17; human septin complexes | line 44; septin filaments | line 45-46; human octameric septin complexes

© 2023. Published by The Company of Biologists under the terms of the Creative Commons Attribution License (https://creativecommons.org/licenses/by/4.0/).

- Results: section title may need adjusting (example:"Human septin filaments self-assemble on lipid model membranes")
- Line 2; human septin octamers | line 11; an enhanced | line 15; septin polymerization | line 16; higher order filament assemblies | line 18; resolution | line 23; septin filaments | line 33; septin-membrane interactions | line 34; septin meshes
- section title may need edition (example: "Septin filament networks on solid surface supported micrometric undulated memranes")
- page 7: "Besides, we never observed any additional third protein layer on top of the described orthogonal mesh, suggesting that all available septin-septin contacts must be implicated in building the two-layer mesh, even at high septin concentrations."
- It should be specified up to which concentrations the previous statement is correct.
- Section "Micrometric membrane reshaping by polymerization of human septins octamers."
- Line 9; septin-GFP octamers

Reviewer 2

Advance summary and potential significance to field

The work by Nakazawa et al. titled 'A specific mesh-like organization of human septin octameric complex drives membrane reshaping and curvature sensitivity' studies the organisation of purified human septin octamers on lipid membranes into higher order mesh-like structures. The authors find that these two-layered septin meshes have the ability to deform the membrane of giant unilamellar vesicles from a smooth into a rippled surface with local bumps and valleys of high local curvature. These findings are accompanied by Monte-Carlo simulations of the interaction of filament networks that have a spontaneous curvature with a deformable 3D membrane leading to similarly rippled surfaces when the filaments have a sufficiently high bending rigidity.

The overall study and its findings are interesting and provide new insights into the specific effects that mammalian/ human septins can have compared to the more commonly studied budding yeast or drosophila septins. While a recent study reported that drosophila septins form bundles and layers, the present study reports that septins tend to form a mesh-like structure of perpendicularly stacked filaments when anchored to lipid bilayers at sufficiently high concentrations. Though this is of potentially high interest to the scientific community, I am afraid that the manuscript in its current form lacks care and control experiments to satisfy the high standards of JCS.

Comments for the author

Main comments/ concerns:
-The authors claim that they have investigated the assembly and organisation of human septin filaments on nm to micron length scales. However, the present work only thoroughly tested assembly of septins on SLBs with a fixed curvature C = 3 micron^(-1) and on LUVs where conclusion of septin organisation and membrane curvature is very limited due to the sample treatment required for electron microscopy.

- how do septins organise on a planar SLB?
-The authors should provide gels of the purified septin proteins.
-it is not very clear whether the concentrations are based on measurements of the octamer concentration or on the concentration of individual septins.
-The model assumes a two-layer septin model, and one can understand that the perpendicular arrangement of filaments with a preferred curvature can lead to the formation of a 'bumpy' GUV. However, this would look differently if there is a single septin layer (similar to the problem studied in the previous work Kumar et al. 2019). It would be good to show simulations of surface deformations by a single layer of septins.
-The experiments show 'bumpy' GUVs at 36 nM septin, for which the SLB experiments suggest that septins do not form a 2 layer network. How do the authors explain this?
-The description of the Monte-Carlo model and the energy calculations could be improved and clarified. It is unclear to me, how a the septin filament can have meaningful bending rigidity perpendicular to its orientation. The authors correctly set the spontaneous curvature $c_{\perp} = 0$, but I

© 2023. Published by The Company of Biologists under the terms of the Creative Commons Attribution License (https://creativecommons.org/licenses/by/4.0/).

think, they should also set $\kappa_\perp = 0$. The simulations should also include the control case of a single septin layer.
-The cause of the nematic order is not clearly described. The parameter epsilon is very phenomenological. What does it represent in the real world and what is the consequence of filament spacing for vesicle deformation? The schematic suggests the septin spacing is due to direct interaction between septins. The authors should add experiments with septin constructs that lack this lateral interaction site.

Minor comments:
Page 5: the phrase ‘Figure 1 displays …’ can be removed and be replaced by ‘(Fig. 1).
Could the authors specify what they mean by ‘in most cases, orthogonal…’, does this mean in most of the LUVs or in most parts of the LUV? A quantification would be helpful (number of LUV with mesh divided by all observed LUVs).
Don’t use a full phrase to refer to a figure. It is sufficient to simply use Page 6: replace ‘Our observations are presented in Figure 2’ with (Fig. 2)
- you mention concentrations ranging from 10-100 nM, but then follow with low concentration (8 nM), then simply write ranging from 8 – 90 nM instead.

Page 7:
-in the phrase ‘above 44 nM septin concentrations…’ why do you say ‘already’?

Page 9,
-Fig 4, you should show also some examples of the irregular GUV deformations.
- how many independent experiments for the measurement of septin rings?

page 13: ‘filaments are closer ‘ to ‘ another…’; ‘the first septin’ layer (?) ‘
should be…’
page 14: the discussion can be made a bit more clear, one phrase, the authors highlight the model needs revisiting because human septins organise in a more complex network-like manner, then they mention that the sensitivity to micrometric curvatures is shared. It is also to highlight that meshwork-like septin organisation was reported in EMs on the cytokinetic ring of budding yeast, including by some of the authors (Bertin & Nogales, 2012).
-Sup fig. 5: provide a better resolution image, the current image quality is very strongly compressed and makes it hard to appreciate any detail. Legend should detail what top and bottom images show, why showing a zoom? From where is the zoom taken?
-Fig 4 and Sup fig 8, the images of GUV and the presented measurements don’t seem to match well. The GUVs shown seem to have peak to peak distances of 2-3 microns (maybe a reference to sup fig 10 should be included in the figure legend), but the data plot shows distances of up to 12 microns or more. The drawbacks of the method to measure peak to peak distance should be discussed. From how many experiments were the vesicles taken? Each experiment should be run at least thrice to ensure reproducibility.
-Sup fig 9: clearly indicate in the figure legend what a) is and what b):
‘Transmission electron microscopy images of (a) human septins octamer-GFP in 50 mM Tris pH 8 containing 300 mM KCl, and, (b) human septins octamer-GFP after 1h incubation in 10 mM Tris pH 7.8 containing 75 mM NaCl.’

-Sup fig 10: something went wrong with the y axis. The length (theta) cannot be >70 micron for a vesicle that has a circumference of 110 micron. Sup table 1: It isn’t clear how common the two different distribution patterns at 26 nM Septin are. Are both patterns observed in the same sample? What is the area fraction showing the two septin filament distributions, e.g. twice as many areas are showing curvature independent septin organisation compared to curvature dependent septin organisation. The number of areas considered for analysis should be rather equal. Why is there such an increase in number of regions analysed between 8.7 nM, 26 nM and 26 nM (2)?

-Sup material page 16: ‘valley’ not ‘vallay’.
The nomenclature could be done better. It can be confusing to use for the global curvature of the ideal sphere H = 1/R = delta (not sure why you call it delta), and then use ‘h’ for the local radius of a hill or valley.

© 2023. Published by The Company of Biologists under the terms of the Creative Commons Attribution License (https://creativecommons.org/licenses/by/4.0/).

- It would be great if the authors could include the preprint by Martins et al. (https://doi.org/10.1101/2022.02.23.4811653) in the discussion. Here human septin octamers are shown to associate with actin filaments in straight lines and apparently mainly flat topographies. Meshworks are not observed. Also not in the reconstitution on SLB where septin concentration is 300 nM. Placing this work in context to the authors manuscript would be good.

Reviewer 3

Advance summary and potential significance to field

In this work Nakazawa and colleagues explore how human septins sense curvature and deform membranes in a variety of in vitro assays. To date, the majority of in vitro work with septin proteins has been done with yeast septins. Recently methods to purify recombinant human septins have been published, allowing the authors to repeat some of their previous assays with these proteins from a different species. Surprisingly, they report a number of behaviors in human septins that differ from yeast septins. At low concentrations both human and yeast septins preferentially align in the concave valleys of the wavy surfaces and also parallel to the surface of the ridges (where there is zero curvature). At higher concentrations, yeast septins pack tightly like a nematic liquid crystal, reinforcing this curvature preference. However, human septins at high concentrations appear to organize orthogonal to each other, creating a cross-hatch type pattern. Smaller differences are seen when yeast and human septins are added to the outside of GUVs. Yeast septins deform the membrane to create spikes on the surface, while human septins create dimples on the GUV surface. They also present a monte carlo based model to describe their findings, treating each septin filament as a director field which interacts either with the underlying membrane, or between the two layers of filaments.

There is a value in highlighting the differences between yeast and mammalian septins, as they clearly have at least slightly different features. At the moment, however, this is mainly an observational study.

Comments for the author

To really make the claims that this meshlike organization drives membrane reshaping and curvature sensitivity I think it would be necessary to do the following:

1) use wavy substrates of different curvature to show how changes in curvature affect this organization forming.

2) break down the effect of septins on GUVs of different sizes and show that curvature impacts the ability to reshape the membrane

3) Make the model more quantitative beyond simply showing images (In both Figures 3&5). In particular some sort of quantitative exploration of the phase space with the parameters would be useful in showing how the system can be tuned between different states, and the dependence on the parameter values (even if they're relative values to each other). This would help move the model from purely describing the experimental results, to potentially predicting changes in organization in specific regimes that could then be tested experimentally (potentially in a future manuscript).

Other suggestions to consider:
- Much is made of the idea that septins are forming two individual layers, but it doesn't seem clear that they are indeed separate. From the images in Fig 1C,D and the supplemental movie, it looks like they're often in the same plane and could easily be interwoven. What is the evidence that they are in fact separate layers? And does it matter for the interpretation? What happens if you increase the concentration even further?

- On the GUVs, the deformations seen with human septins resemble a dimpled surface, whereas with yeast septins spikier protrusions could be seen. These two shapes seem very similar, differing

© 2023. Published by The Company of Biologists under the terms of the Creative Commons Attribution License (https://creativecommons.org/licenses/by/4.0/).

only in the extent of the deformation. If the dimples were deeper it seems like they would form similar spiky projections. Thus, could this difference in membrane deformation tell us anything about the curvature sensing of the human septins vs yeast? Or alternatively, about the strength of the membrane/septin interaction? And it is related potentially to the difference seen in the previous reports of human septin hexamers creating larger deformations?

- There are also a few assumptions that are either baked into the model, or left out that should be justified. First, the septins are given an intrinsic curvature. While I believe I have heard speculation that the octomer itself has some sort of curvature, no citation or reference is given for that assumption. This should be clarified as either an assumption or given a citation. Second the representation of the two layers as director field neglects the filament formation that is clearly going on in the images. While these recombinant septins do not appear to bundle side to side, they are clearly making longer filaments, presumably by annealing end to end. This would incur an energy penalty specifically in the vortex looking structures. Is there any consideration into incorporating longer length scale interactions beyond simply the nearest neighbor vortices and how this would affect the ordering? Third, I don't understand the justification for modeling the increase in septin concentration as simply increasing the septin-membrane interaction term. Fourth the model assumes that there is interaction between the second layer and the membrane, but wouldn't that be screened by the first septin layer? Are the coiled-coiled extensions long enough to still interact with the membrane? And does the second layer of septins alter the membrane interaction with the first layer?

- In the cartoon figure, the coiled-coil extensions from Sept2,6,7 are shown pointing in multiple directions, and essentially being very flexible. How realistic is this? It's also unclear how this orientation of filaments would preclude adding additional layers. For instance in the cartoon you could imagine a third layer being built up using the sept6/7 extensions from either of the horizontal filaments.

Minor points:
- As someone who has not seen thousands of cryo-EM tomography images, some additional annotation on the images in Figure 1 would be helpful for readers. Using the same color for all septins in the first membrane contacting layer (instead of green in the top and middle rows in the column on the right) and defining what is shown in magenta in that middle column.

- In Figure 2 it's hard to tell where the peaks and troughs are in the wavy substrates. It would be helpful to have a side view cartoon above each panel that has the contour to orient the image.

- In figure 4, labels on the images numbered 1-9 in panel B would be helpful since there are multiple different planes, projections and two different labels.

First revision

Author response to reviewers' comments

We thank the reviewers for their global positive and helpful comments which improved our study and manuscript. Following their advices, we have performed additional experiments and analysis of the data to answer the referee's concerns. Specifically, all of the referees were wondering how septin filaments would behave on membranes with various curvatures. Hence, we designed additional wavy substrates covered with lipids displaying various curvatures from flat to highly curved and analyzed the results by Scanning Electron Microscopy. Moreover, additional simulations were carried out to strengthen your arguments. The modifications applied to the manuscript are highlighted in red and new figures have been added.

Reviewer 1 Advance Summary and Potential Significance to Field:

In this study, Nakazawa et al. describe the structural organization of human septins on

© 2023. Published by The Company of Biologists under the terms of the Creative Commons Attribution License (https://creativecommons.org/licenses/by/4.0/).

membranes and examine their curvature sensitivity. With a wide set of techniques that span the study of this protein from the nanometer to the micron scale, the authors revealed the distribution of septin filaments into a 2-layer mesh of orthogonally ordered filaments and studied their curvature sensitivity and effect on free standing membranes. This is a very intriguing observation, as the studies on yeast have shown a contrasting assembly on these filaments. The model for network assembly they propose here is an interesting contribution to the field and opens many questions on the membrane reshaping capabilities of these proteins on reconstituted lipid membranes.

Reviewer 1 Comments for the Author:
The techniques and model together provide good understanding of how these filaments assemble and with which concentration thresholds deformations are enabled, this in a system that has not been sufficiently studied previously. The tomography also adds good evidence of superposed filaments and potential membrane deforming.

However, it seems that the curvature sensing is not defined very clearly and the range of the described specificity seems unclear. It may be useful to detect this behavior in different ranges of curvatures to build a stronger biophysical understanding of these interesting networks. SUV extrusion or tube pulling may be methods of choice to acquire this information.

In previous studies, we and others have highlighted a septin specificity for micrometric curvatures (Beber et al., 2019, Nature Communications, Cannon et al, 2019, JCB.) both in vitro and in situ as opposed to nanometric curvatures. Nonetheless, most curvature sensing proteins like Bar proteins are sensitive to nanometric curvatures and their behaviour can be tested using tube pulling assays or SUVs which dimensions are within tens of nanometers. Conversely, septins are sensitive to micrometric curvatures. When incubated with highly curved membranes (SUV displaying nanometric curvatures), septins straight filaments deform the membranes into flat geometries. Regarding septins, assays probing nanometric curvatures are thus not adapted. We have modified and added an additional sentence in the introductory section to make this point more explicit to the reader.

The data displayed in Figure 2 were collected on substrates with a maximum curvature of 3 μm^{-1} on the upper region of the substrate. We had chosen this specific curvature because it fitted with the range of curvatures resulting from the deformation of Giant Unilamellar Vesicles (see Figure 4). To answer the referee's concern about testing different curvatures, we have indeed carried out additional experiments using wrinkled substrates displaying either shallower (max 1.7 μm^{-1}) or sharper curvatures (max 4.5 μm^{-1}) which led to a new figure (Figure 3). The organization of septins bound to a flat substrate was also assayed (Figure 3.A) Onto the flat substrates, septin filaments do not show any curvature preference. They do not align within any specific orientation and thus appear mostly disorganized (Figure 3A). However, bound to sharp curvatures (4.5 μm^{-1}, Figure 3.C), or low curvature (1.7 μm^{-1}, Figure 3.B) at high septin density, septin filaments appear lying flat on top of positively curved bumps and bend negatively to connect consecutive bumps. Hence, on sharp curvatures septin filaments behave similarly to our prior observations at 3 μm^{-1}. We have analyzed the orientation of septin filaments for all the curvatures tested. The resulting graphs (Figure 3.B and C, lower panels) shows that the orientation of the filaments remains similar for all of the curved substrates with variations in the width of the distribution.

In addition, we have analyzed the membrane deformations induced by septins on GUVs of different diameters to test for any possible role of the initial membrane curvature upon septin addition. The result is shown in Supplementary Figure 10.B and suggests that the deformations are independent on the dimensions (amplitude and periodicity) of the GUVs diameter. Besides, while revisiting the GUVS reshaping we have realized that we had added a systematic error within our matlab script which results in a smaller periodicity in the observed deformations. We have now corrected Figure 3.C to make our analysis correct.

It would be helpful to provide supplementary evidence that one layer interacts with both, membrane and the second layer, but that the second layer only interacts with the first layer.

© 2023. Published by The Company of Biologists under the terms of the Creative Commons Attribution License (https://creativecommons.org/licenses/by/4.0/).

Additionally, a comment on how the second layer composed of the same septin subunits may achieve this selective binding seems to be appropriate.

This point was commented in the discussion section but certainly not sufficiently. Hence, additional explanations were inserted for clarity. From the cryo-tomograms and specifically movies displaying one z slice at a time in a 3D volume, we observe that the two sets of orthogonal septin filaments localize at distinct distances form the surface of liposomes. Hence, one layer interacts primarily with the membrane while a second one binds to the first set of septin filaments. However, one cannot exclude that the upper septin layer interacts as well, to some extent, with the membrane. In addition, from the simulations, one single septin layer of filament is sufficient to induce the observed membrane deformation and one can thus suspect that the second layer might be used to stabilize the first one. Our experimental observations are thus in perfect agreement with our Monte Carlo simulations. Besides that, observations from X ray crystallography, centered on the coiled coil domains, suggest that the septin-septin interaction could result from sept2 coiled-coiled mediated interaction.

There is no mention of the behavior of septins on flat surfaces such as supported lipid bilayers for example. It would be helpful to compare such experiments with the results in Figure 2.

We have performed additional experiments and now display a SEM image of septins bound to a flat surface (Figure 3.A). It does not show a specific orientation of the parallel sheets of septin filaments patterned into a nematic like ordering. This thus suggest that a specific arrangement of septins might be favoured by their interaction with specific curvatures.

Specific major points
-The Result section necessitates further editing, the authors should work on sentence structure and punctuation to ease reading.

We have had our manuscript read by a native speaker to improve the editing and corrected the minor points listed below

-Figure 1 is missing panel labels that the result section mention.

Thanks for noticing this. We have now added those.

-Are there any mesh configuration differences in SUVs that present deformations when compared to septin meshes on spherical SUVs? Are those deformations correlated to the size of the SUV? It would be interesting to yield a deeper analysis on the segmentation performed on those deformed SUVs as confinement and different vesicle curvature might induce changes in filament orientation or distribution.

The SUVs mentioned above are certainly LUVs. SUVs would measure tens of nm in diameters and would not accommodate septin filaments. The curvature of LUVs is not favorable for septin interaction. Only 50 % have septins bound and thus display peculiar shapes (see 2D image below (left) as an example). The organization of septins bound is visible as a mesh-like pattern, when performing 3D reconstruction. As mentioned in the manuscript, the distance between filaments was measured at 18 ± 15 nm. Bound to LUVs (from about 100 nm to 1μm in diameter), septins almost systematically flatten vesicles into “pancake” like liposomes so that they can avoid being positively curved (published in Beber et al 2019) or aggregate the smaller liposomes together within septin bundles (see image below (right) to show an example).

© 2023. Published by The Company of Biologists under the terms of the Creative Commons Attribution License (https://creativecommons.org/licenses/by/4.0/).

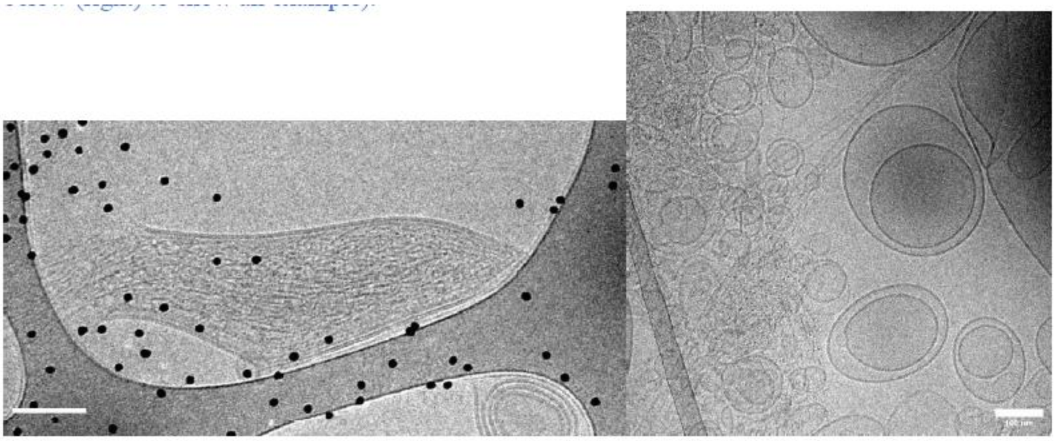

-Can the authors provide information on the distribution of filaments on different surface curvatures? As human septins show a very different organization than the ones from budding yeast, further assessment of their intrinsic curvature should be performed with other patterned structures.

As mentioned above, we have performed experiments on additional substrates: flat, slightly curved and sharply curved to compare for different curvatures. We added an additional figure (Figure 3) and discuss the outcome above and in the core of the manuscript. Those additional experiments were indeed useful and enrich our report.

-Figure 4.B-6 shows a 3D reconstruction with visible septin signal in long structures surrounding the GUV. Is the protein inducing membrane tubulations in those areas? Or is it the result of the membrane being osmotically deflated and therefore showing unspecific protein binding? This should be checked by a negative control.

Reviewer 1 is right. On this image, we do see fluorescent signal extending from the vesicles. This should have been described in the manuscript. We are now thus describing the nature of this protruding signal. This signal stands in the green channel which indicates the presence of GFP labelled septins. Indeed, septins, tend to bundle when their density increase. Being recruited by GUVs, septins accumulate at the surface of vesicles and start bundling from those into hairy - looking filaments. Given the lipid signal is absent within those protrusions we can conclude those are septins only and do not reflect any lipid tubulation.

-The coarse grain models support the observations of the two-layers mesh organization and fit the experimental data but they do not provide valuable information to aid on the assembly of the model proposed.

Note that, both on the wavy substrate and on the vesicle, the orthogonal filaments organization of the 2nd layer with respect to the 1st one, is not an emergent property of our model. It is the result of an explicit interaction term which energetically favours such an orthogonal arrangement. But the pattern of orientation of the 1st layer of filaments on the membrane surfaces, both for the rigid wavy substrate and the deformable vesicle surface, are emergent properties of the model. In addition, the pattern of vesicle deformation in the 2nd case is also an emergent property.

Further, the kinetics of the filament assembly is not provided by our simulation. This is a standard limitation of any Monte-Carlo simulation which cannot address the real time dynamics and real-time self-assembly of filaments. We can only address the final pattern of filaments from a free energy minimization point of view.

Minor points

We thank reviewer 1 for suggesting this alternative title and modified the original one

© 2023. Published by The Company of Biologists under the terms of the Creative Commons Attribution License (https://creativecommons.org/licenses/by/4.0/).

accordingly. In addition, we corrected all of the identified mistakes listed below.

- The title may need tweaking (example: “A human curvature sensitive septin octamer complex drives membrane deformation with a specific mesh-like organization”).
- Introduction: line 3; actin and microtubules | line 6; cell motility and cell compartmentalization |
line 8; Septin mis-regulation | line 17; human septin complexes | line 44; septin filaments | line 45-46; human octameric septin complexes
- Results: section title may need adjusting (example:”Human septin filaments self-assemble on lipid model membranes”)
- Line 2; human septin octamers | line 11; an enhanced | line 15; septin polymerization | line 16; higher order filament assemblies | line 18; resolution | line 23; septin filaments | line 33; septin- membrane interactions | line 34; septin meshes
- section title may need edition (example: “Septin filament networks on solid surface supported micrometric undulated memranes”)
- page 7: “Besides, we never observed any additional third protein layer on top of the described orthogonal mesh, suggesting that all available septin-septin contacts must be implicated in building the two-layer mesh, even at high septin concentrations.”
- It should be specified up to which concentrations the previous statement is correct.
- Section “Micrometric membrane reshaping by polymerization of human septins octamers.”
- Line 9; septin-GFP octamers

Reviewer 2 Advance Summary and Potential Significance to Field:
The work by Nakazawa et al. titled ‘A specific mesh-like organization of human septin octameric complex drives membrane reshaping and curvature sensitivity’ studies the organisation of purified human septin octamers on lipid membranes into higher order mesh-like structures. The authors find that these two-layered septin meshes have the ability to deform the membrane of giant unilamellar vesicles from a smooth into a rippled surface with local bumps and valleys of high local curvature. These findings are accompanied by Monte-Carlo simulations of the interaction of filament networks that have a spontaneous curvature with a deformable 3D membrane leading to similarly rippled surfaces when the filaments have a sufficiently high bending rigidity.
The overall study and its findings are interesting and provide new insights into the specific effects that mammalian/ human septins can have compared to the more commonly studied budding yeast or drosophila septins. While a recent study reported that drosophila septins form bundles and layers, the present study reports that septins tend to form a mesh-like structure of perpendicularly stacked filaments when anchored to lipid bilayers at sufficiently high concentrations. Though this is of potentially high interest to the scientific community, I am afraid that the manuscript in its current form lacks care and control experiments to satisfy the high standards of JCS.

We thank reviewer 2 for his positive comments and thank him for suggesting additional experiments which have benefited to the quality of our study and manuscript.

Reviewer 2 Comments for the Author: Main comments/ concerns:
-The authors claim that they have investigated the assembly and organisation of human septin filaments on nm to micron length scales. However, the present work only thoroughly tested assembly of septins on SLBs with a fixed curvature C = 3 micron^(-1) and on LUVs where conclusion of septin organisation and membrane curvature is very limited due to the sample treatment required for electron microscopy.

Most curvature sensitive proteins usually recognize nanometric sharp curves that lead to endocytosis or exocytosis events for instance. However, septins have been shown to be sensitive to micrometric curvatures, both in vitro and in situ, which is rather unusual, when considering curvature sensing proteins. Hence we have focused our study on micrometric curvatures. We have performed additional new experiments on wrinkled patterns displaying either shallow (1.7 um^{-1}) or sharp (4.5 um^{-1}) curvatures as a comparison with our original set of data performed using

© 2023. Published by The Company of Biologists under the terms of the Creative Commons Attribution License (https://creativecommons.org/licenses/by/4.0/).

substrates of 3 um^{-1} curvatures. The results are displayed in a new figure (Figure 3) and are commented in the manuscript and above (see answer to reviewer 1, first point). In addition, we have analyzed the behaviour of GUVs displaying a range of diameters both experimentally and using simulations (see Figure 6-B). We have thus concluded (Figure-6B) that the membrane deformations observed on GUVs are independent on the vesicles diameters.
Concerning LUVs, imaged by cryo-EM, their dimensions only allow for the flattening of vesicles which become “pancake-like”. Observed nanometric deformation for higher concentrations are in agreement with the curvature preference observed with our wavy substrate.

- how do septins organise on a planar SLB?
We have performed experiments on planar SLBs and now display the result in Figure 3.A. On a bilayer deposited on a flat surface, septins organize in a nematic fashion with rather random orientations .

-The authors should provide gels of the purified septin proteins.
We now provide a gel of the GFP septins used within this study at all stages throughout purification (see Supplementary Figure 12). The observed pattern is similar to the expected migration of septins on a SDS gel.

-it is not very clear whether the concentrations are based on measurements of the octamer concentration or on the concentration of individual septins.

The concentrations (molar) are given considering the octamer concentrations. We added a mention in the text to make this point more clear.

-The model assumes a two-layer septin model, and one can understand that the perpendicular arrangement of filaments with a preferred curvature can lead to the formation of a ‘bumpy’ GUV. However, this would look differently if there is a single septin layer (similar to the problem studied in the previous work Kumar et al. 2019). It would be good to show simulations of surface deformations by a single layer of septins.

According to our model the bumpy structure is caused by the membrane-septin interaction of the 1st layer (Figure 5D and E). If the 2nd layer interacts with the membrane equally strongly then the bumpy structure is annulled (Figure 5A). Therefore, even without the 2nd layer bumpy structure (Figure D and E) will remain identical. Hence, we can assume that the role of the second layer might be to stabilize the first layer of septins. Septins in the 2nd layer indeed seem to passively orient perpendicular to the septins in the 1st layer but do not deform the membrane.

-The experiments show ‘bumpy’ GUVs at 36 nM septin, for which the SLB experiments suggest that septins do not form a 2 layer network. How do the authors explain this?

Again, the deformations are supposedly induced by the first layer of septin filaments. Therefore, the bumpy structure observed on GUVs at 36 nM is expected.

-The description of the Monte-Carlo model and the energy calculations could be improved and clarified. It is unclear to me, how a the septin filament can have meaningful bending rigidity perpendicular to its orientation. The authors correctly set the spontaneous curvature $c_{\perp} = 0$, but I think, they should also set $\kappa_{\perp} = 0$. The simulations should also include the control case of a single septin layer.

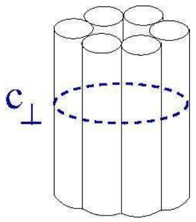

Note that the bending rigidity is that of the membrane not this of septin filaments. Such a

© 2023. Published by The Company of Biologists under the terms of the Creative Commons Attribution License (https://creativecommons.org/licenses/by/4.0/).

rigidity term is used when septins form bundle and forces the membrane to adhere to it to form a cylinder of radius $=1/c_{\perp}$ This was explored in Kumar et al , 2019. In fact, the spiky structures on vesicles formed due to Yeast septin can be explained by this bundling effect where $c_{\perp}$ is positive. However, in the context of human septins bundling effect is absent or weak, and thus, $c_{\perp}=0$. Yet, $\kappa_{\perp}$ is not null because the membrane gets unstable along the perpendicular direction if zero curvature ($c_{\perp}=0$) is not imposed along this direction. In fact with this we can ignore the overall kappa term which ensures a baseline isotropic bending elasticity.

-The cause of the nematic order is not clearly described. The parameter epsilon is very phenomenological. What does it represent in the real world and what is the consequence of filament spacing for vesicle deformation? The schematic suggests the septin spacing is due to direct interaction between septins. The authors should add experiments with septin constructs that lack this lateral interaction site.

In the physics of liquid crystal, the parameter epsilon stands for the elastic constants for splay and bend (in a one parameter approximation) and its net effect is to align the nematic rods. In the context of septin filaments, here, epsilon promote parallel alignment of the filaments. Parallel alignment is generic to many cytoskeletal filamentous like actin, microtubule. This is why cytoskeletal dynamics of cell cortex is effectively modelled by active nematics. In the same spirit, we use the nematic term with epsilon for septin ordering. Moreover, this parallel ordering tendency must depend on the distance between two filaments i.e., on surface coverage (density) of septins. We can therefore reflect a higher septin density by increasing the interaction strength with epsilon.

Minor comments:
Page 5: the phrase ‘Figure 1 displays …’ can be removed and be replaced by ‘(Fig. 1).
We have corrected this.

Could the authors specify what they mean by ‘in most cases, orthogonal…’, does this mean in most of the LUVs or in most parts of the LUV? A quantification would be helpful (number of LUV with mesh divided by all observed LUVs).

We actually meant “in most of the LUVs”. We have now added quantification.

Because the curvature of LUVs are nanometric, the interaction with septins is not favorable. Hence only a proportion of the LUVs have septins bound and are thus deformed into flattened vesicles. About 50% have septins bound and can be extracted from looking at their features at low magnification. All of the analyzed liposomes (n = 7) had orthogonal meshes bound.
We have now added the number of LUVs and tomograms that were analyzed.

Don’t use a full phrase to refer to a figure. It is sufficient to simply use

Page 6: replace ‘Our observations are presented in Figure 2’ with (Fig. 2)
- you mention concentrations ranging from 10-100 nM, but then follow with low concentration (8 nM), then simply write ranging from 8 – 90 nM instead.
We have modified the text accordingly. The correct concentrations are from 8 to 87 nM.

Page 7:
-in the phrase ‘above 44 nM septin concentrations…’ why do you say ‘already’?
We mean that we get a full density at 44 nM septin concentrations while at 30 nM the septin density remained rather scarced. It seems that there is a threshold between 30 and 44 nM where full coverage of septins occurs, on the surface. Indeed, no density intermediate could ever be spotted.
We have thus modified the manuscript to make this point clearer.

Page 9,
-Fig 4, you should show also some examples of the irregular GUV deformations.

We show an example in supplementary Figure 7.B. It is explicitly indicated now, in the text.

© 2023. Published by The Company of Biologists under the terms of the Creative Commons Attribution License (https://creativecommons.org/licenses/by/4.0/).

- how many independent experiments for the measurement of septin rings?

Three independent experiments were carried out. This piece of information has now been added.

page 13: ‘filaments are closer ‘ to ‘ another…’; ‘the first septin’ layer (?) ‘ should be…’
page 14: the discussion can be made a bit more clear, one phrase, the authors highlight the model needs revisiting because human septins organise in a more complex network-like manner, then they mention that the sensitivity to micrometric curvatures is shared. It is also to highlight that meshwork-like septin organisation was reported in EMs on the cytokinetic ring of budding yeast, including by some of the authors (Bertin & Nogales, 2012).

We modified the discussion accordingly.

-Sup fig. 5: provide a better resolution image, the current image quality is very strongly compressed and makes it hard to appreciate any detail. Legend should detail what top and bottom images show, why showing a zoom? From where is the zoom taken?

The lower panel shows an image collected at a higher magnification. It is thus not a zoom of the upper panel.
We have checked for the images resolution. The filamentous pattern is quite dense and make individual filaments less discernible than on samples with a lower septin density, though.

-Fig 4 and Sup fig 8, the images of GUV and the presented measurements don’t seem to match well. The GUVs shown seem to have peak to peak distances of 2-3 microns (maybe a reference to sup fig 10 should be included in the figure legend), but the data plot shows distances of up to 12 microns or more. The drawbacks of the method to measure peak to peak distance should be discussed. From how many experiments were the vesicles taken? Each experiment should be run at least thrice to ensure reproducibility.

We thank to the reviewers, for noticing this. We indeed found some errors in our Matlab scripts used to extract the deformation dimensions when we started revisiting this particular question. We now provide a new figure 4C and Supplementary figure 10.
In terms of reproducibility, the results from different concentration of 4 independent experiments were used for this analysis, and regular plus irregular bumpy deformation of all spherical deformed GUV was taken into account for this analysis. Figure 4C provides full information of regular and irregular bumpy distances, thus having a long tail in high values, however, clearly showing a large population around 1.8 um (mean + sd 1.1um).

-Sup fig 9: clearly indicate in the figure legend what a) is and what b):
‘Transmission electron microscopy images of (a) human septins octamer-GFP in 50 mM Tris pH 8 containing 300 mM KCl, and, (b) human septins octamer-GFP after 1h incubation in 10 mM Tris pH 7.8 containing 75 mM NaCl.’

We have modified the legend accordingly. Thanks for noticing this.

-Sup fig 10: something went wrong with the y axis. The length (theta) cannot be >70 micron for a vesicle that has a circumference of 110 micron.

Thanks for pointing this out. We corrected the errors that was within our Matlab script. Please find a modified sup. Figure 10.

Sup table 1: It isn’t clear how common the two different distribution patterns at 26 nM Septin are. Are both patterns observed in the same sample? What is the area fraction showing the two septin filament distributions, e.g. twice as many areas are showing curvature independent septin organisation compared to curvature dependent septin organisation. The number of areas considered for analysis should be rather equal. Why is there such an increase in number of regions analysed between 8.7 nM, 26 nM and 26 nM (2)?

© 2023. Published by The Company of Biologists under the terms of the Creative Commons Attribution License (https://creativecommons.org/licenses/by/4.0/).

Indeed, both patterns can be visualized in the same sample. Actually, 26 nM – 30 nM is close to the concentration threshold where the protein density becomes maximum and the surface gets fully covered. This is most likely why at this specific concentration range, several filamentous self- organization can be observed.
The sample at low septin concentration (8.7 nM) is not fully covered by septins at all. It is about the concentration above which septins start to interact with the membrane (see phase diagram, figure 5.A). Hence there were much less areas to be analyzed in that case.

-Sup material page 16: ‘valley’ not ‘vallay’.
This has been corrected.

The nomenclature could be done better. It can be confusing to use for the global curvature of the ideal sphere H = 1/R = delta (not sure why you call it delta),and then use ‘h’ for the local radius of a hill or valley.

Actually, the curvature of the patterns do not only depend in a radius but on both the periodicity Λ and the amplitude A of the wavy patterns following the formula: $C=4*\pi*A/\Lambda^2$.

- It would be great if the authors could include the preprint by Martins et al. (https://doi.org/10.1101/2022.02.23.481653) in the discussion. Here human septin octamers are shown to associate with actin filaments in straight lines and apparently mainly flat topographies. Meshworks are not observed. Also not in the reconstitution on SLB where septin concentration is 300 nM. Placing this work in context to the authors manuscript would be good.
This is indeed a great suggestion and we have now included it in the discussion as well

Reviewer 3 Advance Summary and Potential Significance to Field:
In this work Nakazawa and colleagues explore how human septins sense curvature and deform membranes in a variety of in vitro assays. To date, the majority of in vitro work with septin proteins has been done with yeast septins. Recently methods to purify recombinant human septins have been published, allowing the authors to repeat some of their previous assays with these proteins from a different species. Surprisingly, they report a number of behaviors in human septins that differ from yeast septins. At low concentrations both human and yeast septins preferentially align in the concave valleys of the wavy surfaces, and also parallel to the surface of the ridges (where there is zero curvature). At higher concentrations, yeast septins pack tightly like a nematic liquid crystal, reinforcing this curvature preference. However, human septins at high concentrations appear to organize orthogonal to each other, creating a cross- hatch type pattern. Smaller differences are seen when yeast and human septins are added to the outside of GUVs. Yeast septins deform the membrane to create spikes on the surface, while human septins create dimples on the GUV surface. They also present a monte carlo based model to describe their findings, treating each septin filament as a director field which interacts either with the underlying membrane, or between the two layers of filaments.

There is a value in highlighting the differences between yeast and mammalian septins, as they clearly have at least slightly different features. At the moment, however, this is mainly an observational study.

We thank reviewer 3 for noticing that we highlight here the differences observed between yeast and mammalian septins. However, given we provide Monte Carlo simulations to interpret and understand the observed pattern we believe our study extends beyond being observational only. We hope that the new version of our manuscript and its additional analysis and experiments will be convincing enough.

Reviewer 3 Comments for the Author:
To really make the claims that this meshlike organization drives membrane reshaping and curvature sensitivity I think it would be necessary to do the following:

Actually we claimed that membrane reshaping is done solely by the 1st layer of septin and not the orthogonal mesh which includes the 2nd layer. In our simulation when both the layers

© 2023. Published by The Company of Biologists under the terms of the Creative Commons Attribution License (https://creativecommons.org/licenses/by/4.0/).

interact with the membrane in equal strengths their effects neutralize each other and the vesicle maintains its undeformed spherical shape. We showed this by systematically reducing the interaction strength between the 2nd layer and the membrane relative to that between the 1st layer and the membrane, see Figure 5. We have added additional comments to make this point more clear.

1) use wavy substrates of different curvature to show how changes in curvature affect this organization forming.

This was a request from reviewers 1 and 2 and we have thus performed additional experiments following this line (see Figure 3). Our conclusions are summarized in the first point to the response of reviewer 1.

Note that a single wavy substrate offers continuously varying curvature spanning from high negative value in the concave troughs to high positive value on the convex hills, passing smoothly through zero curvature along the channels. Note that diagonal orientations of septins in the troughs and the hills have smaller value of curvature (negative and positive, respectively). Therefore, a filament has the liberty to scan different curvatures by rotating itself in the local tangent plane. To examine possible orientations, it is equivalent to, a) keep the substrate curvature same and change the intrinsic curvature of septin (which we have done across Figure 2), or b) vary the substrate curvature keeping the intrinsic curvature of septin same.

2) break down the effect of septins on GUVs of different sizes and show that curvature impacts the ability to reshape the membrane

We have performed additional simulations to probe how the GUV size (and hence its curvature) affects the deformation structure when septin is put in. The results are shown in supplemantary- Figure.6-B,where we report deformed GUVs for three different sizes. Essentially, the periodicity of the bumps does not vary with the GUV's diameter. The total number of bumps scale with the area of the GUV keeping distance between the bumps approximately same. Experimentally, we have compared the periodicity of our deformations with the radii of the GUVs and essentially saw no significant correlation between those parameters (Supplementary Figure 10.B).

3) Make the model more quantitative beyond simply showing images (In both Figures 3&5). In particular some sort of quantitative exploration of the phase space with the parameters would be useful in showing how the system can be tuned between different states, and the dependence on the parameter values (even if they're relative values to each other). This would help move the model from purely describing the experimental results, to potentially predicting changes in organization in specific regimes that could then be tested experimentally (potentially in a future manuscript).

We have done additional simulations exploring the shape space as a function of the anisotropic membrane rigidity $\kappa_{||}$ and septin curvature $C_{||}$. A phase diagram of shapes is shown in Figure 6-C. Buds and tubes are seen to form at low $\kappa_{||}$ and $C_{||}$ values. Rationalization of these shapes are detailed in the corresponding figure caption.

Other suggestions to consider:
- Much is made of the idea that septins are forming two individual layers, but it doesn't seem clear that they are indeed separate. From the images in Fig 1C,D and the supplemental movie, it looks like they're often in the same plane and could easily be interwoven. What is the evidence that they are in fact separate layers? And does it matter for the interpretation? What happens if you increase the concentration even further?

From the supplementary movie describing consecutive slices in z within the 3D reconstruction, we indeed see that the two orthogonal sets of filaments lie at different distances form the membrane, implying that they are separate layers. We have added additional sentences to point out this outcome. Actually, as suggested in the discussion we assume that the protein - membrane

© 2023. Published by The Company of Biologists under the terms of the Creative Commons Attribution License (https://creativecommons.org/licenses/by/4.0/).

and protein-protein interaction are mediated through different sets of C-terminus domains and more particularly coiled coils. Hence, the orthogonal sets of filaments are not separated per se, remaining somehow connected. With increasing density as observed by SEM, it is possible that the interaction is stronger given the connecting domains are all involved in the interaction. We have now enriched the discussion to explain our views.

- On the GUVs, the deformations seen with human septins resemble a dimpled surface, whereas with yeast septins spikier protrusions could be seen. These two shapes seem very similar, differing only in the extent of the deformation. If the dimples were deeper it seems like they would form similar spiky projections. Thus, could this difference in membrane deformation tell us anything about the curvature sensing of the human septins vs yeast? Or alternatively, about the strength of the membrane/septin interaction? And it is related potentially to the difference seen in the previous reports of human septin hexamers creating larger deformations?

The spiky membrane structures seen for Yeast septins breaks the in-out symmetry and we believe these are due to formation of narrow septin bundles. Conversely, the wavy membrane deformations on GUVs caused by human septins preserve the in-out symmetry. Quantitatively speaking, the curvature undergoes a discontinuity at a spike whereas over a smooth hill it changes continuously. However the periodicity of these structures are nearly same (~ 4 nm) in both cases, which is because the curvature and width of the valleys (distance between two maxima) is set by the negative intrinsic curvature of septins, a property which could be universal across yeast and human.

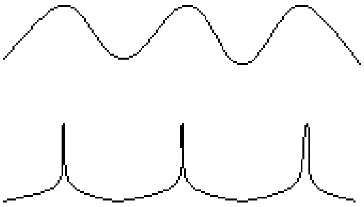

- There are also a few assumptions that are either baked into the model, or left out that should be justified. First, the septins are given an intrinsic curvature. While I believe I have heard speculation that the octomer itself has some sort of curvature, no citation or reference is given for that assumption. This should be clarified as either an assumption or given a citation.

Indeed, recent cryo-EM studies from the Garratt lab, performed on a human septin complexes, suggests that the septin rod displays a kink. We have thus added a reference in the text (Mendonça et al., JMB, 2021) and cited it to insist on the possible septin intrinsic curvature.

Second, the representation of the two layers as director field neglects the filament formation that is clearly going on in the images. While these recombinant septins do not appear to bundle side to side, they are clearly making longer filaments, presumably by annealing end to end. This would incur an energy penalty specifically in the vortex looking structures. Is there any consideration into incorporating longer length scale interactions beyond simply the nearest neighbor vortices and how this would affect the ordering?

Note that a strong epsilon (εLL) in our model not only promotes parallel nematic ordering laterally, but it also aligns the filaments which are neighbours along their long axes. This builds up orientational correlation over longer length scales, which is equivalent to having long polymers. But our simulation being a Monte-Carlo simulation it cannot take care of the actual temporal dynamics of poly/depolymerization.

On the wavy substrates the vortex looking structure results from mismatch between the intrinsic curvature of septin and the curvature of the maximally curved direction in the concave valleys.

© 2023. Published by The Company of Biologists under the terms of the Creative Commons Attribution License (https://creativecommons.org/licenses/by/4.0/).

In course grained models like ours each nematic rod represents many filaments with an average direction. So interaction between only neighbouring vertices does not mean microscopically a filament only interacts with its neighbouring filament. Furthermore, long range orientational correlations can arise via local interactions between neighbouring filaments as explained above. Therefore, enhancing the range of interaction in coarse grained models only stabilize the ordering patterns over longer length scales but does not change it qualitatively.

Further, note that the effect of the filaments, whether long or short, is to bend the membrane locally. If the local curvature imposed by per unit length of a filament is fixed, few longitudinally aligned filaments cumulatively achieve the same total bending effect as what a longer filament achieves.

Third, I don’t understand the justification for modeling the increase in septin concentration as simply increasing the septin-membrane interaction term.

We have explained this already in our reply to Referee-2.

Fourth, the model assumes that there is interaction between the second layer and he membrane, but wouldn’t that be screened by the first septin layer? Are the coiled-coiled extensions long enough to still interact with the membrane? And does the second layer of septins alter the membrane interaction with the first layer?

Yes we indeed assumed that the interaction between the second layer and the membrane is screened by the 1st layer. Effect of gradually higher degree of screening is shown in Figure 5.

- In the cartoon figure, the coiled-coil extensions from Sept2,6,7 are shown pointing in multiple directions, and essentially being very flexible. How realistic is this? It’s also unclear how this orientation of filaments would preclude adding additional layers. For instance in the cartoon you could imagine a third layer being built up using the sept6/7 extensions from either of the horizontal filaments.

This remains rather speculative. Indeed, the long coiled coils are long enough so they could point in other directions. We have generated a new scheme which is now more realistic.

Minor points:
- As someone who has not seen thousands of cryo-EM tomography images, some additional annotation on the images in Figure 1 would be helpful for readers. Using the same color for all septins in the first membrane contacting layer (instead of green in the top and middle rows in the column on the right) and defining what is shown in magenta in that middle column.

We have added some “guidelines” in green in the image to highlight portions of the filaments. Indeed, cryo-EM generates images with low signal over noise ratio and some features are sometimes challenging to discern.

- In Figure 2 it’s hard to tell where the peaks and troughs are in the wavy substrates. It would be helpful to have a side view cartoon above each panel that has the contour to orient thetimage.

Reviewer 3 is right.
To solve this issue, we have added arrows on Figure 2 to point at “hills” (positive curvatures) on each image.

- In figure 4, labels on the images numbered 1-9 in panel B would be helpful, since there are multiple different planes, projections and two different labels.

We have added labels with septin concentrations to enhance the clarity of the figure.

© 2023. Published by The Company of Biologists under the terms of the Creative Commons Attribution License (https://creativecommons.org/licenses/by/4.0/).

Second decision letter

MS ID#: JOCES/2022/260813

MS TITLE: A human curvature sensitive septin octamer complex drives membrane deformation with a specific mesh-like organization

AUTHORS: Koyomi Nakazawa, Gaurav Kumar, Brieuc Chauvin, Aurélie Di Cicco, Luca Pellegrino, Michael Trichet, Bassam Hajj, João Cabral, Anirban Sain, Stephanie Mangenot and Aurelie Bertin
ARTICLE TYPE: Research Article

We have now reached a decision on the above manuscript.

To see the reviewers' reports and a copy of this decision letter, please go to: https://submit-jcs.biologists.org and click on the 'Manuscripts with Decisions' queue in the Author Area. (Corresponding author only has access to reviews.)

As you will see, the reviewers gave favourable reports. However, reviewer 2 has raised some points concerning clarification of the text. It is easier to do this now rather than at the proof stage. I hope that you will be able to carry these out because I would like to be able to accept your paper once you return it.

Please ensure that you clearly highlight all changes made in the revised manuscript to address reviewer 2's comments. Please avoid using 'Tracked changes' in Word files as these are lost in PDF conversion.

I should be grateful if you would also provide a point-by-point response detailing how you have dealt with the points raised by the reviewers in the 'Response to Reviewers' box. Please attend to all of the reviewers' comments. If you do not agree with any of their criticisms or suggestions please explain clearly why this is so.

Reviewer 1

Advance summary and potential significance to field

This is a solid study that advances our understanding of septin organization and function in well-controlled membrane environments. After revisions, it is acceptable for JOCES.

Comments for the author

The authors have made significant changes in the manuscript which corrected for errors on Figures, data analysis and writing. On the one hand, the design of additional curved topographies and the new simulations performed support the authors' understanding of septin filament assembly and behaviour and allow for a clear comparison of experimental conditions to draw the pertinent conclusions. Additionally, they have addressed the concerns regarding septin accumulation in GUVs and provided information on septin bundling due to local accumulation of the protein on their surface. On the other hand, sentence structure and overall writing has improved and the discussion section now contains a clearer and more coherent interpretation of the data. Given these changes, the revised manuscript can be considered fit for publication.

© 2023. Published by The Company of Biologists under the terms of the Creative Commons Attribution License (https://creativecommons.org/licenses/by/4.0/).

Reviewer 2

Advance summary and potential significance to field

The revised version of the article is much improved and the points raised by the reviewers were addressed in a satisfactory manner. However, the quality and clarity of the text and the figures can be improved in parts of the manuscript that are outlined below.

Comments for the author

Introduction:
The second phrase stands in contrast to the statement of the previous phrase, and it is not clear what the first phrase is supposed to mean: 'The behavior of human septins appears universal, particularly on membranes. Indeed, human septin complexes are often interacting with membranes displaying micrometer scale membrane curvatures (Cannon et al., 2017). ' Clearer would be a formulation like: Human septins can play many important roles in cells, particularly when associated to membranes. Indeed, septin complexes are …'

Results:

'Write 'model lipid membranes' instead of 'lipid model membranes'.

'on all deformed vesicles by septins' is grammatically not correct. Do you mean 'on all deformed vesicles containing septins…' or 'on all vesicles deformed by septins…'?

'The first filament layer (closest to the membrane) appears at t = 2 seconds and t = 31 seconds, within the sequence of images displayed in the movie, while the second layer appears at t = 3 and 29 seconds, suggesting that the 2 filamentous layers are at distinct distance from the membrane' : you refer to the time in the movie to talk about positions in the z-stack, this is not very good. Better is to directly refer to the z-planes that you measured, e.g. '…appears at z-slices 2 and 31… the second layer appearing at z-slices 3 and 29, this would correspond to a distance of xx micron taking into account the distance between two z-slices. '

the phrase 'We have tested curvatures of 1.7 µm-1, 3 µm-1 and 4.5 µm-1 at the maximum, at the top of the wavy substrates' is a bit unclear. Better and easier could be the following: 'We have tested wavy substrates with peak curvatures of 1.7 µm-1, 3 µm-1 and 4.5 µm-1.'

'bound to flat curvatures…' it is clear what is meant, but it is unprecise language (happens several times in the text). Septins don't bind to curvatures, they bind to membranes. Simply write 'Bound to flat membranes, septins…'

'the positively curved domains of the "waves" ' it is ok to talk about domains when talking about regions of septins showing nematic order, but it would be good to rather use 'positively curved areas of the waves' when describing the substrate.

'Hence the lengths of the filaments incorporated within those domains are shorter': this statement is difficult to put into context. There is no earlier mention about septin filament lengths. That higher substrate curvatures impose a stronger obstacle for septins to orient randomly, leading to a longer range alignment, is not surprising.

H_||^i¤ c_||^ . Did you forget to enter something in the exponent (empy box)? Or should you simply use the c with a subscript and no super script?

'using GUVs that would be "floppy", with a lower bending modulus such inwards invagination were not observed ': why not writing simply 'using GUVs with membranes of low bending modulus such inwards…' because GUVs can also be floppy when their membrane tension is low, independent of the bending modulus.

'sensitivity to ensure conserved properties over species.' : again the wording. Who is ensuring anything? A more appropriate wording could be something along the lines of : '… curvature sensitivity indicating that these properties are conserved between species'.

Discussion:

'Drosophila or human hexemeric septins' -> hexameric

Methods:

The equations in the Model for the monte carlo simulations have to be formatted. Ideally using the program latex.

© 2023. Published by The Company of Biologists under the terms of the Creative Commons Attribution License (https://creativecommons.org/licenses/by/4.0/).

Figures:

Figure 1, the alignment of image panels and zoom boxes is very sloppy. In A, the green lines to guide are not very convincing as they don't seem to overlay well with the contrast images. The zoom box is mal-positioned (the pattern of dots does not correspond to the pattern in the original region). The same holds for B, where the zoom is not showing the patterns depicted in the original box. Are the green lines hand drawn or result of some segmentation? Some lines are red, does this have any meaning?

The SEM images suggest that there are a lot of membrane defects and excess membranes. Those might disturb longer range septin organisation. It would be good if either the authors comment on that possibility or compare organisation in regions with cleaner membranes vs.

Reviewer 3

Advance summary and potential significance to field

In this revision, Nakazawa and colleagues have expanded their findings on the differences between human and yeast septins and their ability to sense membrane curvature.

Comments for the author

The authors have satisfactorily addressed my comments from their initial submission.

Second revision

Author response to reviewers' comments

Dear Editor,

We were glad to obtain positive comments and feedback from our revised manuscript.

We have adressed the remaining concerns from reviewer 2 which had noticed wording, typo and mistakes that had escaped our attention.

The latest corrections are added in blue in the text.

We hope this novel version will be considered for publication in Journal of Cell Science. Best regards
Aurélie Bertin

We thank the referees for their positive feedback and acknowledgement of our efforts to revise the first version of our manuscript. We have addressed the additional comments from reviewer 2 and modified our manuscript accordingly. The most recent revisions appear in blue in the text.

Reviewer 1 Advance Summary and Potential Significance to Field:

This is a solid study that advances our understanding of septin organization and function in well-controlled membrane environments. After revisions, it is acceptable for JOCES.

Reviewer 1 Comments for the Author:

The authors have made significant changes in the manuscript which corrected for errors on Figures, data analysis and writing. On the one hand, the design of additional curved topographies and the new simulations performed support the authors' understanding of septin filament assembly and

© 2023. Published by The Company of Biologists under the terms of the Creative Commons Attribution License (https://creativecommons.org/licenses/by/4.0/).

behaviour and allow for a clear comparison of experimental conditions to draw the pertinent conclusions. Additionally, they have addressed the concerns regarding septin accumulation in GUVs and provided information on septin bundling due to local accumulation of the protein on their surface. On the other hand, sentence structure and overall writing has improved and the discussion section now contains a clearer and more coherent interpretation of the data. Given these changes, the revised manuscript can be considered fit for publication.

We thank reviewer 1 for revising our manuscript again and considering the points we have addressed.

Reviewer 2 Advance Summary and Potential Significance to Field:

The revised version of the article is much improved and the points raised by the reviewers were addressed in a satisfactory manner. However, the quality and clarity of the text and the figures can be improved in parts of the manuscript that are outlined below.

Reviewer 2 Comments for the Author: Introduction:
The second phrase stands in contrast to the statement of the previous phrase, and it is not clear what the first phrase is supposed to mean: 'The behavior of human septins appears universal, particularly on membranes. Indeed, human septin complexes are often interacting with membranes displaying micrometer scale membrane curvatures (Cannon et al., 2017). ' Clearer would be a formulation like: Human septins can play many important roles in cells, particularly when associated to membranes. Indeed, septin complexes are …'

Thanks for spotting this unclear statement. We modified this section.

Results:

Write 'model lipid membranes' instead of 'lipid model membranes'.

It was found and corrected 3 times in the text.

'on all deformed vesicles by septins' is grammatically not correct. Do you mean 'on all deformed vesicles containing septins…' or 'on all vesicles deformed by septins…'?

This was changed to "on all vesicles deformed by septins" for a correct grammar and meaning.

'The first filament layer (closest to the membrane) appears at t = 2 seconds and t = 31 seconds, within the sequence of images displayed in the movie, while the second layer appears at t = 3 and 29 seconds, suggesting that the 2 filamentous layers are at distinct distance from the membrane' : you refer to the time in the movie to talk about positions in the z-stack, this is not very good. Better is to directly refer to the z-planes that you measured, e.g. '…appears at z-slices 2 and 31… the second layer appearing at z-slices 3 and 29, this would correspond to a distance of xx micron taking into account the distance between two z-slices.

Thanks for pointing this out. We kept the time indication in the movie so that the reader can spot easily when one set of filament appears after the other. Besides, we added indications in Z distances (12 nm between each sets of filaments) as suggested by reviewer 2.

the phrase 'We have tested curvatures of 1.7 μm-1, 3 μm-1 and 4.5 μm-1 at the maximum, at the top of the wavy substrates' is a bit unclear. Better and easier could be the following: 'We have tested wavy substrates with peak curvatures of 1.7 μm-1, 3 μm-1 and 4.5 μm-1.'

Thanks for the suggestion.

'bound to flat curvatures…' it is clear what is meant, but it is unprecise language (happens several times in the text). Septins don't bind to curvatures, they bind to membranes. Simply write 'Bound to flat membranes, septins…'

Indeed, this is better.

© 2023. Published by The Company of Biologists under the terms of the Creative Commons Attribution License (https://creativecommons.org/licenses/by/4.0/).

‘the positively curved domains of the “waves” ‘ it is ok to talk about domains when talking about regions of septins showing nematic order, but it would be good to rather use ‘positively curved areas of the waves’ when describing the substrate.

We corrected this as well.

Hence the lengths of the filaments incorporated within those domains are shorter’: this statement is difficult to put into context. There is no earlier mention about septin filament lengths. That higher substrate curvatures impose a stronger obstacle for septins to orient randomly, leading to a longer range alignment, is not surprising.

The sentence has been modified and refer to micrometric long filaments for higher curvatures, to make things clearer: “Hence the lengths of the filaments incorporated within those smaller domains are shorter and are about 500 nm long as opposed to a few microns for higher curvatures.(…) This suggests that substrates of higher curvatures impose the orientation of septin filaments at a larger scale, imposing a stronger obstacle for septins to orient randomly.”

H_‖|^i¤ c_‖^. Did you forget to enter something in the exponent (empy box)? Or should you simply use the c with a subscript and no super script?

This should actually be empty and it has been corrected.

‘using GUVs that would be “floppy”, with a lower bending modulus such inwards invagination were not observed ‘: why not writing simply ‘using GUVs with membranes of low bending modulus such inwards…’ because GUVs can also be floppy when their membrane tension is low, independent of the bending modulus.

This is correct. We have modified this as suggested.

‘sensitivity to ensure conserved properties over species.’ : again the wording. Who is ensuring anything? A more appropriate wording could be something along the lines of : ‘… curvature sensitivity indicating that these properties are conserved between species’.

Indeed we modified this.

Discussion:

‘Drosophila or human hexemeric septins’ -> hexameric

Thanks for noticing this typo error.

Methods:

The equations in the Model for the monte carlo simulations have to be formatted. Ideally using the program latex.

Thanks for noticing this issue. We formatted the equations properly.

Figures:

Figure 1, the alignment of image panels and zoom boxes is very sloppy. In A, the green lines to guide are not very convincing as they don’t seem to overlay well with the contrast images. The zoom box is mal-positioned (the pattern of dots does not correspond to the pattern in the original region). The same holds for B, where the zoom is not showing the patterns depicted in the original box. Are the green lines hand drawn or result of some segmentation? Some lines are red, does this have any meaning?

We have aligned all of the panels more precisely and readjusted the zoom panels so that they coincide at best with the original enlarged region of the images. Also we made sure the guide lines

© 2023. Published by The Company of Biologists under the terms of the Creative Commons Attribution License (https://creativecommons.org/licenses/by/4.0/).

were actually superimposed with septin filaments, which was not perfectly performed. Also we can not highlight all of septin filaments, which would make the images confusing. Hence only portions of some of the filaments were highlighted.

The SEM images suggest that there are a lot of membrane defects and excess membranes. Those might disturb longer range septin organisation. It would be good if either the authors comment on that possibility or compare organisation in regions with cleaner membranes vs.

Indeed as noticed, liposomes are still and consistently present in our sample even though we rinse samples thoroughly after the deposition of lipid bilayers. We believe that liposomes, still in solution, might bind to septin filaments already interacting with lipid bilayers. Indeed, naked bilayers barely display any additional vesicles. We thereby assume that rinsing them off completely is not achievable. We added a sentence in the manuscript to comment on this.

Reviewer 3 Advance Summary and Potential Significance to Field:

In this revision, Nakazawa and colleagues have expanded their findings on the differences between human and yeast septins and their ability to sense membrane curvature.

Reviewer 3 Comments for the Author:

The authors have satisfactorily addressed my comments from their initial submission.

We thank reviewer 3 for taking the time to consider our revised document and giving positive feedback.

Third decision letter

MS ID#: JOCES/2022/260813

MS TITLE: A human curvature sensitive septin octamer complex drives membrane deformation with a specific mesh-like organization

AUTHORS: Koyomi Nakazawa, Gaurav Kumar, Brieuc Chauvin, Aurélie Di Cicco, Luca Pellegrino, Michael Trichet, Bassam Hajj, João Cabral, Anirban Sain, Stéphanie Mangenot and Aurelie Bertin
ARTICLE TYPE: Research Article

I am happy to tell you that your manuscript has been accepted for publication in Journal of Cell Science, pending standard ethics checks.

© 2023. Published by The Company of Biologists under the terms of the Creative Commons Attribution License (https://creativecommons.org/licenses/by/4.0/).
